# Phenotypic and genotypic characterization of *Staphylococcus aureus* strains isolated from otitis externa: Emergence of CC30-*spa* t019-SCC*mec* IV carrying PVL as major genotype

**DOI:** 10.1016/j.heliyon.2024.e32002

**Published:** 2024-05-28

**Authors:** Zahra Rahmani, Sareh Sadat Hosseini, Parmida Bagheri, Masoud Dadashi, Mehrdad Haghighi, Mehdi Goudarzi

**Affiliations:** a Department of Otorhinolaryngology, Loghman Hospital, Shahid-Beheshti University of Medical Sciences, Tehran, Iran; b Department of Microbiology, School of Medicine, Shahid Beheshti University of Medical Sciences, Tehran, Iran; c Department of Biotechnology, Faculty of Life Sciences and Biotechnology, Shahid Beheshti 7 University, Tehran, Iran; d Department of Microbiology, School of Medicine, Alborz University of Medical Sciences, Karaj, Iran; e Department of Infectious Diseases, Imam Hossein Teaching and Medical Hospital, Shahid Beheshti University of Medical Sciences, Tehran, Iran

**Keywords:** Biofilm formation, *Staphylococcus aureus*, Otitis externa, Antibiotic resistance, SCC*mec*, *Spa*

## Abstract

The increasing emergence of *Staphylococcus aureus* as the primary causative agent of otitis externa has been noted; however, detailed information regarding the molecular characteristics of these strains in Iran remains scarce. The current study aims to investigate both genotypic and phenotypic attributes of *S. aureus* strains implicated in ear infections. In the present work, we analyzed 60 *S. aureus* strains isolated from cases of otitis externa over a period of 45 months. The resistance patterns were determined using disk diffusion and microbroth dilution methods. All *S. aureus* isolates were confirmed by the *nucA* polymerase chain reaction assay, and their biofilm production was assessed by a microtiter plate assay. Molecular characterization of the isolates was performed using the staphylococcal cassette chromosome *mec,* multilocus sequence typing, and staphylococcus protein A typing methods. Overall, the results indicated that 44 out of 60 *S. aureus* isolates (73.3 %) were methicillin-resistant *Staphylococcus aureus*. Resistance to mupirocin and vancomycin was observed in 13.3 % and 1.7 % of the tested isolates, respectively. Furthermore, out of the 60 *S. aureus* isolates, 56 strains (93.4 %) were classified as positive biofilm strains at different levels. Twelve distinct clonal lineages were identified. The vast majority of *S. aureus* isolates belonged to CC30/ST30-MRSA IV/t019 (41.7 %). Among the 31 strong biofilm producers, the majority (64.5 %) belonged to CC30/ST30-MRSA IV/t019 clone. Biofilm negative isolates belonged to CC22/ST22 (2 isolates), CC8/ST585 (one isolate), and CC8/ST8 (one isolate). Our result revealed that about three-quarters of PVL-positive strains belonged to CC30/ST30. Our data confirmed the presence of MSSA strains among CC30/ST30 and CC22/ST22 isolates. The mupirocin resistant isolates (n = 8) belonged to CC8/ST585-MRSA III/t713 (37.5 %), CC8/ST239-MRSA III/t030 (25 %), CC8/ST8-MRSA IV/t008 (12.5 %), CC8/ST239-MRSA III/t037 (12.5 %), and CC22/ST22-MRSA IV/t790 (12.5 %) lineages. The VRSA strain belonged to the CC8/ST8-MRSA IV/t008 lineage, carrying the *vanA* determinant. iMLS_B_ phenotypes (n = 14) were distributed across different lineages, including CC30/ST30-MRSA IV/t019 (21.5 %), CC30/ST30-MSSA/t021 (21.5 %), CC22/ST22-MSSA/t005 (14.3 %), CC8/ST239-MRSA III/t030 (14.3 %), CC22/ST22-MSSA/t1869 (7.1 %), CC22/ST22-MRSA IV/t790 (7.1 %), CC8/ST239-MRSA III/t037 (7.1 %), and CC1/ST772-MRSA IV/t10795 (7.1 %). These findings highlight significant genotypic diversity and high biofilm formation among our isolates. The frequent occurrence of the CC/ST30 clone in *S. aureus* strains isolated from otitis externa reflects the emergence of these lineages as a predominant clone in Iran, posing a significant public health concern.

## Highlights

• First genetic variability study on *S. aureus* strains isolated from patients with otitis externa in Iran.
• Genetic diversity of MRSA strains with a CC30-*spa* t019-SCC*mec* IV carrying PVL as major lineage was observed.
• The strong presence of HLMUPR MRSA isolates belonged to CC8/ST585-MRSA III/t713 clone.
• The high ability of CC30/ST30 strains in generation of biofilm at strong level was observed.

## Abbreviations

OE: Otitis externa
OM: Otitis media
PCR: Polymerase Chain Reaction
CLSI: Clinical and Laboratory Standards Institute
LLMUPR: Low-Level Mupirocin Resistant
HLMUPR: High-Level Mupirocin Resistant
MIC: Minimum Inhibitory Concentration
VISA: Vancomycin-Intermediate *S. aureus*
VRSA: Vancomycin-Resistant *S. aureus*
SD: Standard Deviation
OD: Optical Density
PCI: Phenol-Chloroform Isoamyl Alcohol
SDS: Sodium Dodecyl Sulfate
BLAST: Basic Local Alignment Search Tool
NCBI: National Center for Biotechnology Information
SCC*mec*: Staphylococcal Cassette Chromosome *mec*
VNTR: Variable Number of Tandem Repeats
MLST: Multilocus Sequence Typing
CC: Clonal Complex
ST: Sequence Type
SPSS: Statistical Package for the Social Sciences

## 1 Introduction

Ear infections are considered a significant public health concern, impacting both children and adults, with an anticipated increase in their incidence. Ear infections can be either acute or chronic conditions, including otitis externa (OE) and otitis media (OM), with OE being associated with the external auditory canal [1]. In most cases of OE, timely detection of infectious agents and prompt treatment can reduce symptoms to a considerable extent, leading to recovery within a few days. However, the condition may persist for several months or longer for some of the patients [1,2]. Literature has indicated that approximately 350 million people worldwide have experienced at least one ear infection, with over half of them suffering from significant hearing loss [1,3]. Several microorganisms, including Gram-negative bacteria, such as *Proteus mirabilis, Klebsiella pneumonia, Escherichia coli,* and *Serratia marcescens*, are known to be responsible for OE. Recent evidence suggests that *Staphylococcus aureus* and *Pseudomonas aeruginosa* are the most common causative agents of OE [3,4]. In recent decades, the increasing occurrence and concurrent resistance to multiple drugs in S. *aureus* strains isolated from OE have emerged as a major concern in healthcare settings worldwide, raising significant challenges [1,2]. While recent studies have focused on understanding the molecular types, antibiotic resistance and biofilm patterns of *S. aureus* isolated from OE cases in Iran, there remains a palpable gap in knowledge regarding the genetic diversity and biofilm formation of this bacterium. Accordingly, this study attempted to identify the biofilm production, antibiotic resistance genes, and genetic diversity of *S. aureus* isolated from OE cases.

## 2 Material and methods

### 2.1 Ethics statement

This research has obtained the approval of the Ethics Committee of Shahid Beheshti University of Medical Sciences in Tehran, Iran (IR.SBMU.MSP.REC.1400.764). Verbal consent was obtained from all participants.

### 2.2 *S. aureus* identification

This study was conducted between March 2019 and November 2022 at two teaching hospitals affiliated with Shahid Beheshti University of Medical Sciences. For OE cases, purulent discharges were collected using a sterile swab. The inclusion criteria included samples from OE patients who hadn't received antibiotics for at least three weeks prior to their clinical visit and had no recent hospitalizations. Exclusion criteria applied to those who had undergone antibiotic therapy three weeks before the study or had recent hospital admissions within. Patient specimens were immediately cultured on blood agar plates (HiMedia, Mumbai, India) and incubated at 37 °C for 24 h. The golden or white colonies, consisting of gram-positive cocci in the form of clusters, were identified through the evaluation of catalase (HiMedia, Mumbai, India) and coagulase (HiMedia, Mumbai, India) production using the tube method with rabbit-citrated plasma. For further analysis, the colonies were then cultivated on mannitol-salt agar (HiMedia, Mumbai, India) and DNase mediums (HiMedia, Mumbai, India). The genetic confirmation of isolates was achieved through the detection of the *nuc*A gene using polymerase chain reaction (PCR) with forward and reverse primer sequences of F: GCGATTGATGGTGATACGGTT and R: AGCCAAGCCTTGACGAACTAAAGC [5]. PCR was performed in a total volume of 25 μl, containing 2 μL of DNA template (0.5 μg), 5 μL of reverse and forward primers (1 μM), 12.5 μL PCR mix (SinaClon, Tehran, Iran), and 5.5 μL distilled water. The DNA thermocycler (Eppendorf, Hamburg, Germany) was programmed with the following temperature settings: an initial denaturation step was carried out at 95 °C for 4 min, followed by 35 cycles consisting of 94 °C denaturation for 45 s, 55 °C annealing for 45 s, and 72 °C extension for 60 s. The program concluded with a final elongation step for 5 min at 72 °C. Afterwards, the PCR products were electrophoresed on 1.5 % agarose gels. Confirmed *S. aureus* isolates were cultured in tryptic soy broth (HiMedia, Mumbai, India) containing 20 % sterile glycerol and were stored at −70 °C.

### 2.3 Determination of isolates susceptibility

The antibiotic susceptibility of isolates was determined using the Kirby-Bauer method, adhering to the Clinical and Laboratory Standards Institute (CLSI) criteria for antibiotic resistance pattern (CLSI 2022). The tested antibiotics included ciprofloxacin (CIP), clindamycin (CLI), erythromycin (ERY), fusidic acid (FUS), gentamicin (GEN), linezolid (LIN), nitrofurantoin (NIT), penicillin (PEN), rifampin (RIF), and tetracycline (TET). Additionally, susceptibility to vancomycin and mupirocin (including low-level (LLMUPR) and high-level (HLMUPR) mupirocin resistance) was confirmed using the broth microdilution method. According to the 2022 CLSI guidelines, mupirocin MIC breakpoints were established as follows: LLMUPR at 8–128 μg/ml and HLMUPR at ≥ 256 μg/ml. According to the CLSI guideline, vancomycin-intermediate *S. aureus* (VISA) and vancomycin-resistant *S. aureus* (VRSA) are currently classified based on vancomycin MICs of 4–8 μg/ml and ≥16 μg/ml, respectively. The d-zone test was performed to detect inducible clindamycin resistance (CLSI 2022). Methicillin resistance was evaluated with a cefoxitin disc (30 μg) on Mueller Hinton agar, following CLSI guidelines [6]. Multidrug resistance was defined as the lack of susceptibility to at least one antibiotic in three or more unrelated classes [7]. For quality control, reference strains of *S. aureus* ATCC 25923, ATCC 43300, and ATCC 29213 were included.

### 2.4 Microtiter plate (MtP) assay

The biofilm formation assay was performed as previously described [8]. In this method, a 1:10 dilution of an overnight culture of *S. aureus* in Tryptic Soy Broth (HiMedia, Mumbai, India) supplemented with 1 % glucose (Merck, Germany) was prepared at 37 °C. Individual wells were filled with 200 μL of the dilution before incubation for 24 h at 37 °C under static conditions without shaking. Following incubation, the suspended bacteria were removed by gentle tapping and washing three times with 200 μL phosphate buffer saline (PBS; pH 7.2). Adherent bacteria were fixed with 99 % methanol for 15 min, air-dried for 20 min, and stained with 200 μL safranin solution (0.1 %) for 5 min. Then, the wells were rinsed four times with distilled water to remove excess stains. The adhered bacterial cells were re-solubilized in 200 μL of 33 % (v/v) glacial acetic acid (Merck, Germany) and incubated at 37 °C for 15 min. TSB-supplemented with 1 % glucose was used as the negative control. The optical density (OD) of the stained biofilm was measured at a 490 nm wavelength using an ELISA. Calculation of the mean OD + 3 × standard deviation (SD) of blank control samples yielded an OD cut-off (ODc) value, used in further classifying the strains into four categories based on biofilm production: No biofilm (OD ≤ 0.059), weak (ODc < OD ≤ 2 × ODc), moderate (2 × ODc < OD ≤ 4 × ODc), and strong (OD > 4 × ODc) [8]. The *S. epidermidis* ATCC 35984 strain was used as a positive control for biofilm formation.

### 2.5 Extraction of genomic DNA

Bacterial DNA was extracted from overnight growth on nutrient agar (HiMedia, Mumbai, India) using the phenol-chloroform isoamyl alcohol (PCI) technique [9]. DNA was extracted following the protocol outlined by Sambrook and Russell. A 600 μL TSB culture was transferred to a 2 ml microcentrifuge tube and centrifuged at 14000 rpm for 5 min. The supernatant was then removed, and the pellet was resuspended in 600 μL of nuclease-free water. Subsequently, 10 μL of Proteinase K (250 μg/ml) and 12 μL of 10 % sodium dodecyl sulfate (SDS) (Sigma-Aldrich, St. Louis, Mo) were added, and the mixture was incubated at 56 °C for 1 h. An equal volume of PCI (25:24:1) (600 μL) was added, followed by vortexing for 15 s and centrifugation at 12000 rpm for 10 min. The top aqueous layer was transferred to a new microcentrifuge tube, and 600 μL of PCI was added, vortexed for 15 s, and then centrifuged at 15871×*g* for 15 min. The resulting supernatant was transferred to another tube. Chloroform:isoamyl alcohol was added, vortexed for 15 s, and centrifuged at 12000 rpm for 10 min. The supernatant was transferred to a new tube, following the addition of an equal volume of ethanol (Merck, Germany), isopropanol (Merck, Germany), and one-tenth volume of sodium acetate (Sigma-Aldrich, St. Louis, Mo). The mixture was incubated at −80 °C for 30 min, followed by centrifugation at 10000 rpm for 5 min. The supernatant was carefully removed without disturbing the pellet, which was then washed with 500 μL of 70 % chilled ethanol. After centrifugation at 10000 rpm for 5 min, the supernatant was discarded, and the pellet was dried at room temperature for 10 min before being resuspended in 100 μL of H2O. The DNA was stored at −20 °C until needed. The DNA content and purity were verified using a Nanodrop 2000 spectrophotometer (Thermo Fisher Scientific, Carlsbad, USA).

### 2.6 Detection of mecA and toxin encoding determinants

*S. aureus* strains were evaluated for the presence of the exfoliative toxin, Panton-Valentine leukotoxin, and toxic shock syndrome toxin genes, including *eta*, *etb, pvl*, and *tst* by PCR assay [10]. The presence of *van* and *mec*A-mediated resistance was also detected using PCR as described elsewhere [10]. The primer sequences were also checked by BLAST service available on the National Center for Biotechnology Information (NCBI) GenBank website (https://blast.ncbi.nlm.nih.gov/Blast.cgi) (Table S1). The PCR assay was carried out in a final volume of 25 μL, containing 18 μL of *Taq* DNA polymerase master mix (SinaClon, Tehran, Iran), 5 μL of DNA template (50 ng), and 1 μL of each forward and reverse primers (10 pM). PCR conditions for amplification of toxin-encoding genes by thermocycler (Eppendorf co., Hamburg, Germany) are as follows: initial denaturation for 5 min at 94 °C, 30 cycles of denaturation at 94 °C for 45 s (*eta* and *pvl*), 35 cycles of denaturation at 94 °C for 1 min (*etb* and *tst*), annealing at 56 °C for 45 s (*eta* and *pvl*), annealing at 55 °C for 45 s (*etb* and *tst*), and extension at 72 °C for45 s. The final extension was carried out at 72 °C for 5 min. PCR products were analyzed using electrophoresis on 1 % agarose gel. Also, DNA bands were visualized by staining via ethidium bromide, and photographed under UV illumination. The GeneRuler™ 100 bp Plus DNA Ladder (Fermentas, Vilnius, Lithuania) was used as a molecular size marker.

### 2.7 Genotypic characterization

#### *2.7.1* *SCCmec* (staphylococcal cassette chromosome *mec*) *typing*

Multiplex PCR was employed to identify SCC*mec* types according to the oligonucleotide sequences and conditions described by Boye et al. [11]. Amplification reactions were set up in a 50 μL reaction mixture, comprising of 14 μL of distilled water, 21 μL of *Taq* DNA polymerase 2X master mix (SinaClon, Tehran, Iran), 1 μL of each forward and reverse primers, and 5 μL of DNA template. The reactions were carried out in a thermocycler (Eppendorf, Hamburg, Germany) with the following setting: initial denaturation at 94 °C for 5 min, 35 cycles of denaturation at 94 °C for 45 s, annealing at 56 °C for 45 s, and elongation at 72 °C for 45 s, followed the final cycle of extension at 72 °C for 5 min. The banding patterns obtained were analyzed by comparison with those of reference strains: ATCC 10442 (SCC*mec* type I), N315 (SCC*mec* type II), 85/2082 (SCC*mec* type III), MW2 (SCC*mec* type IVa), and WIS (SCC*mec* type V). Negative DNA control (PCR grade water) was included in all sets of PCR. The PCR products were electrophoresed in a 0.5 % agarose gel containing TBE running buffer at 80 V for 60 min, then photographed under UV light by a gel documentation system (UVItec, UK). The GeneRuler™ 100 bp Plus DNA Ladder (Fermentas, Vilnius, Lithuania) was used as a molecular size marker.

#### 2.7.2 Staphylococcus protein A (spa) typing

According to the method introduced by Harmsen et al. [12], all isolates were genetically classified by *spa* typing with specific primers (Table S1). This method uses the variable number tandem repeat (VNTR) region of the *spa* gene, which consists of repeated units that are typically 24 base pairs (bp) in length (but exceptions of 21–30 exist). To identify *spa* types based on these individual repeat lengths, the PCR-amplified products of the VNTR region were sequenced. During sequencing, the number of 24 bp repeat units in the amplified DNA fragment was determined to make up the *spa* type for a particular *S. aureus* strain. PCR reaction was done in a 25 μL reaction mixture comprising 10 μL of distilled water, 13 μL of *Taq* DNA polymerase master mix (SinaClon, Tehran, Iran), 0.5 μL of each forward and reverse primers, and 1 μL of DNA template in 0.2 mL PCR tubes. The reactions were carried out in a thermocycler (Eppendorf, Hamburg, Germany), with the temperature cycling conditions being as follows: initial denaturation at 95 °C for 10 min, 30 cycles of denaturation at 95 °C for 30 s, annealing at 56 °C for 30 s, and elongation at 72 °C for 45 s, followed by the final cycle of extension at 72 °C for 10 min. Each PCR run included a matching reaction mixture with water as a negative control. The purification of PCR products and nucleotide sequence analysis were perofmed using a commercial kit (QIAquick PCR Purification) and an ABI Prism 377 automated sequencer, respectively (Applied Biosystems, PerkinElmer Co., Foster City, CA). The sequences were edited using Chromas software (Version 1.45, Australia), and *spa* profiles were analyzed by submitting the collected data to the Ridom SpaServer data repository (http://www.spaserver.ridom.de).

### 2.8 Multilocus sequence typing (MLST)

All isolates underwent the MLST assay following the established protocol, described by Goudarzi et al., 2016 [13] (Table S2). This assay included the utilization of primers, PCR reaction conditions, and specific procedures. The identification of allele profiles and sequence types (STs) was achieved by comparing the acquired sequences of housekeeping genes (*tpiA*, *arcC*, *gmK*, *glpF*, *aroE*, *yqiL*, and *pta*) with the information available on the MLST database website (https://pubmlst.org/). The eburst program version 3 was utilized to determine the clustering of related sequence types (STs), referred to as a clonal complex (CC). CC for *S. aureus* is also provided by searching for the ST on PubMLST.

### 2.9 Statistical analysis

Statistical analyses were performed using the Statistical Package for the Social Sciences (SPSS) software version 22 and GraphPad Prism 5 (GraphPad Software, Inc., La Jolla, CA, USA). Chi-squared test or Fisher's exact test was used for calculating the *p* value. A *p* value < 0.05 was considered statistically significant. Chromas software (Version 1.45, Australia) was used for sequence analysis.

## 3 Results

In the present study, 60 *S. aureus* were isolated from 178 OE samples. Among these, 20 isolates (33.3 %) were from male participants, and 40 isolates (66.7 %) were from female participants (*p* value < 0.05), with a mean age of 38 years (ranging from 18 to 69 years). Of the 60 *S. aureus* isolates, 44 (73.3 %) were identified as methicillin-resistant *S. aureus* (MRSA), and the remaining 16 (26.7 %) as methicillin-susceptible *S. aureus* (MSSA). As shown in Table 1, the isolates showed high resistance to penicillin (86.7 %), gentamycin (78.3 %), and tetracycline (75 %). In contrast, the strains exhibited lower resistance to vancomycin (1.7 %), mupirocin (13.3 %), and nitrofurantoin (13.3 %). All tested strains displayed susceptibility to linezolid. Notably, two isolates (3.3 %) tested were susceptible to all tested antibiotics. Our findings revealed that, except for rifampin and fusidic acid, resistance rates to all tested antibiotics were higher in MRSA strains than in MSSA strains. The resistance rate to rifampin was equally distributed (50 %) between MRSA and MSSA strains. However, resistance to fusidic acid was found to be twice as high in MSSA strains compared to MRSA strains. Among the 16 MSSA isolates, none exhibited resistance to nitrofurantoin, vancomycin, or mupirocin. Furthermore, MRSA strains showed resistance to gentamicin and tetracycline at rates nearly three times higher than MSSA, and their resistance to erythromycin and ciprofloxacin in MRSA was nearly four times that of MSSA strains. In total, 56 isolates (93.3 %) were confirmed as multidrug-resistant (MDR) strains. Among the 44 MRSA and 16 MSSA isolates, 95.5 % (42 isolates) and 87.5 % (14 isolates) exhibited an MDR pattern, respectively.

**Table 1:** The resistance rates of MRSA and MSSA strains obtained from OE cases.

Antibiotic^a^	MSSA n (%)	MRSA n (%)	Total n (%)
PEN	8 (15.4)	44 (84.6)	52 (86.7)
GEN	12 (25.5)	35 (74.5)	47 (78.3)
TET	13 (28.9)	32 (71.1)	45 (75)
ERY	8 (19.5)	33 (80.5)	41 (68.3)
CIP	7 (20.6)	27 (79.4)	34 (56.7)
CLI	2 (7.4)	25 (92.6)	27 (45)
RIF	8 (50)	8 (50)	16 (26.7)
FUS	6 (66.7)	3 (33.3)	9 (15)
NIT	0 (0)	8 (100)	8 (13.3)
MUP	0 (0)	8 (100)	8 (13.3)
VAN	0 (0)	1 (100)	1 (1.7)

a ^PEN, Penicillin; CLI, Clindamycin; NIT, Nitrofurantoin; ERY, Erythromycin; TET, Tetracycline; CIP, Ciprofloxacin; MUP, Mupirocin; GEN, Gentamicin; VAN, Vancomycin; RIF, Rifampicin; FUS, Fusidic acid^.

Fourteen different resistance patterns were identified, with the most common being PEN, TET, ERY, CLI, GEN, CIP (20 %; 12/60), followed by PEN, ERY, TET, GEN, CIP (15 %; 9/60), and GEN, TET, RIF, FUS (10 %; 6/60). The susceptibility testing showed that 27 (45 %) and 14 (23.3 %) isolates were confirmed as constitutive clindamycin resistance (cMLS_B_) and inducible clindamycin resistance (iMLS_B_) phenotypes, respectively. Both cMLS_B_ and iMLS_B_ phenotypes were more prevalent in MRSA compared to MSSA strains (41.6 % vs. 3.3 % and 13.3 % vs. 10 %). As presented in Table 1, 13.3 % of isolates were confirmed to be mupirocin-resistant, all exhibiting the HLMUPR phenotype. One vancomycin-resistant *S. aureus* (VRSA) isolate was detected (MIC value of 64 μg/ml), which carried the *vanA* gene. Biofilm formation was observed at various degrees: strong (51.7 %), moderate (23.3 %), and weak (18.3 %), with four isolates (6.7 %) showing no biofilm formation (6.7 %) (Fig. S1). Of the tested isolates, 86.7 % (52 out of 60) were found to be toxigenic. The *pvl* gene was the most commonly detected gene (70 %; 42/60), followed by *eta* gene (11.7 %; 7/60), and *tst* gene (5 %; 3/60) (Figs. S1–4). Among 44 MRSA strains, 35 isolates carried the *pvl* gene (79.5 %) (*p* value < 0.004), 3 isolates (6.8 %) the *tst* gene, and 3 isolates (6.8 %) the *eta* gene. Of the 16 MSSA isolates, 7 (43.8 %) carried the *pvl* gene and 4 (25 %) the *eta* gene.

As depicted in Table 2, the majority of MRSA strains (95.5 %; 42/44) were positive for biofilm formation ability. Among these, 66 % (29/44), 15.9 % (7/44), and 13.6 % (6/44) were strong, weak, and moderate biofilm formers, respectively. Additionally, 4.5 % (2/44) did not form biofilms. In contrast, of the 16 MSSA strains, 7 isolates (43.8 %) produced moderate biofilms, 5 isolates (31.2 %) were weak, 2 isolates (12.5 %) were strong, and 2 isolates (12.5 %) did not produce biofilms.

**Table 2:** Categorization of biofilm production of *S. aureus* strains isolated from OE.

Average OD range^a^	Biofilm degree	Number (%)
>0.942	Strong producer	31 (51.7)
0.495< OD ≤ 0.942	Moderate producer	13 (21.7)
0.310< OD ≤ 0.495	Weak producer	12 (20)
≤0.310	Non-producers	4 (6.6)

a Optical density.

**Fig. 1:**
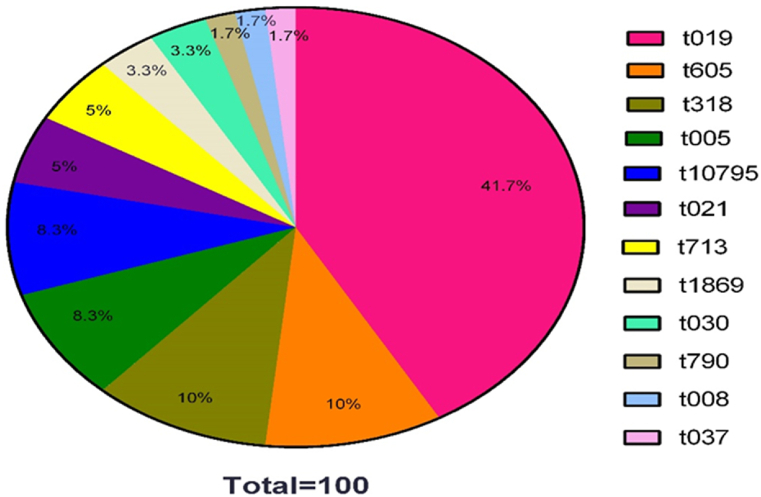
Distribution of *spa* types in *S. aureus* strains isolated from OE cases.

The SCC*mec* typing results revealed that type IV was the most prevalent among MRSA strains, accounting for 86.4 % (38/44), followed by type III (13.6 %; 6/44). The *spa* typing of the 60 isolates recovered from OE cases identified twelve distinct *spa* types among the tested isolates. t019 was the most frequently identified *spa* type identified among isolates (Table S3). The distribution of *spa* types among 60 *S. aureus* isolates is presented in Fig. 1.

As presented in Table 3, isolates were resolved into 4 CCs. All CC30/ST30 isolates were biofilm producers. Three-fourths of CC30/ST30 strains were PVL positive (P < 0.002). The majority of CC22/ST22 isoates were MSSA (87.5 %), and 75 % of these strains were found to carry *pvl* gene. All the CC8/ST239 isolates were found to carry *tst* gene. Our analysis demonstrated the presence of *eta* toxin in CC8/ST585-MRSA III/t713 (5 %), CC30/ST30-MSSA/t318 (3.3 %), and CC22/ST22-MSSA/t1869 (3.3 %). Based on our results, 12 lineages including CC30/ST30-MRSA IV/t019 (41.7 %), CC30/ST30-MRSA IV/t605 (10 %), CC30/ST30-MSSA/t318 (10 %), CC22/ST22-MSSA/t005 (8.3 %), CC1/ST772-MRSA IV/t10795 (8.3 %), CC30/ST30-MSSA/t021 (5 %), CC8/ST585-MRSA III/t713 (5 %), CC22/ST22-MSSA/t1869 (3.3 %), CC8/ST239-MRSA III/t030 (3.3 %), CC22/ST22-MRSA IV/t790 (1.7 %), CC8/ST239-MRSA III/t037 (1.7 %), and CC8/ST8-MRSA IV/t008 (1.7 %) were identified. Allelic profiles and nucleotide sequence among alleles of 7 MLST housekeeping genes of *S. aureus* isolates are presented in Tables S4 and S5. Approximately 90 % of CC22/ST22 and one-fourth of CC30/ST30 strains were MSSA, with all isolates in CC30 being biofilm producers. Mupirocin-resistant isolates were primarily found in CC8/ST585-MRSA III/t713 (37.5 %; 3/8), CC8/ST239-MRSA III/t030 (25 %; 2/8), CC8/ST8-MRSA IV/t008 (12.5 %; 1/8), CC8/ST239-MRSA III/t037 (12.5 %; 1/8), and CC22/ST22-MRSA IV/t790 (12.5 %; 1/8), all exhibiting the HLMUPR phenotype. The VRSA strain belonged to CC8/ST8-MRSA IV/t008 and carried the *vanA* determinant. Isolates with cMLS_B_ phenotype belonged to *spa* type t019-MRSA-IV (18 isolates), t605-MRSA-IV (6 isolates), t005-MSSA (2 isolates), and t008-MRSA-IV (1 isolate). iMLS_B_ strains were distributed across various lineages, including CC30/ST30-MRSA IV/t019 (21.5 %; 3/14), CC30/ST30-MSSA/t021 (21.5 %; 3/14), CC22/ST22-MSSA/t005 (14.3 %; 2/14), CC8/ST239-MRSA III/t030 (14.3 %; 2/14), CC22/ST22-MSSA/t1869 (7.1 %; 1/14), CC22/ST22-MRSA IV/t790 (7.1 %; 1/14), CC8/ST239-MRSA III/t037 (7.1 %; 1/14), and CC1/ST772-MRSA IV/t10795 (7.1 %; 1/14). The results indicated nine fusidic acid-resistant *S. aureus* strains (15 %), which belonged to CC30/ST30-MSSA/t318 (66.7 %; 6/9), CC8/ST239-MRSA III/t030 (22.2 %; 2/9), and CC8/ST239-MRSA III/t037 (11.1 %; 1/9). Overall, molecular typing revealed heterogeneity among the *S. aureus* isolates.

**Table 3:** Characteristics of the 60 *S. aureus* strains obtained from OE cases.

Clonal complex/Sequence type (CC/ST) (n; %)	*spa* type (n; %)	SCC*mec* type (% indicated when not 100 %)	Toxins (n; % indicated when not 100 %)	biofilm formation degree (n; % indicated when not 100 %)	Resistance pattern^a^(n; %)	Total n (%)
CC30/ST30	t019 (25; 62.5)	IV	*pvl* (23; 92)	Strong (20; 80), Moderate (5; 20)	PEN, TET, ERY, CLI, GEN, CIP (9; 36)	40 (66.7)
					PEN, TET, ERY, CLI, RIF, NIT, GEN, CIP (4; 16)	
					PEN, TET, ERY, CLI, RIF, CIP (3; 12)	
					PEN, TET, ERY, CLI, GEN (2; 8)	
					PEN, ERY, TET, GEN, CIP (3; 12)	
					PEN, GEN (2; 8)	
					PEN (2; 8)	
	t605 (6; 15)	IV	*pvl* (5; 83.3)	Strong (4; 66.7), Moderate (2; 33.3)	PEN, TET, ERY, CLI, GEN, CIP (3; 50)	
					PEN, TET, ERY, CLI, RIF, NIT, GEN, CIP (1; 16.7)	
					PEN, TET, ERY, CLI, GEN (2; 33.3)	
	t318 (6; 15)	MSSA	*eta* (2; 33.3)	Strong (2; 33.3), Weak (4; 66.7)	GEN, TET, RIF, FUS (6; 100)	
	t021 (3; 7.5)	MSSA	*pvl* (2; 66.7)	Weak	PEN, ERY, GEN (1; 33.3)	
					PEN, ERY, TET, GEN, CIP (2; 66.7)	
CC22/ST22	t005 (5; 62.5)	MSSA	*pvl*	Moderate (3; 60), Weak (1; 20), No biofilm (1; 20)	PEN, TET, ERY, CLI, RIF, CIP (2; 40)	8 (13.3)
					PEN, ERY, TET, GEN, CIP (2; 40)	
					Without resistance (1; 20)	
	t1869 (2; 25)	MSSA	*eta*	Weak (1; 50), No biofilm (1; 50)	PEN, ERY, TET, GEN, CIP (1; 50)	
					Without resistance (1; 50)	
	t790 (1; 12.5)	IV	*pvl*	Strong	PEN, ERY, GEN, MUP (1; 100)	
CC8/ST585	t713(3; 42.9)	III	*eta*	Weak (2; 66.7), No biofilm (1; 33.3)	PEN, GEN, MUP (3; 100)	7 (11.7)
CC8/ST239	t030 (2; 28.6)	III	*tst*	Weak (1; 50), Strong (1; 50)	PEN, TET, ERY, NIT, CIP, MUP, FUS (2; 100)	
	t037 (1; 14.3)	III	*tst*	Weak	PEN, TET, ERY, NIT, CIP, MUP, FUS (1; 100)	
CC8/ST8	t008 (1; 14.3)	IV	*pvl*	Strong	PEN, TET, ERY, CLI, MUP, VAN (1; 100)	
CC1/ST772	t10795 (5; 100)	IV	*pvl*	Weak (2; 40), Strong (2; 40), No biofilm (1; 20)	PEN, ERY, TET, GEN, CIP (1; 20)	5 (8.3)
					PEN, GEN (4; 80)	

a ^PEN, Penicillin; CLI, Clindamycin; NIT, Nitrofurantoin; ERY, Erythromycin; TET, Tetracycline; CIP, Ciprofloxacin; MUP, Mupirocin; GEN, Gentamicin; VAN, Vancomycin; RIF, Rifampicin; FUS, Fusidic acid^.

PVL-positive strains belonged to CC30/ST30-MRSA IV/t019 (54.7 %; 23/42), CC30/ST30-MRSA IV/t605 (11.9 %; 5/42), CC1/ST772-MRSA IV/t10795 (11.9 %; 5/42), CC22/ST22-MSSA/t005 (11.9 %; 5/42), CC30/ST30-MSSA/t021 (4.8 %; 2/42), CC22/ST22-MRSA IV/t790 (2.4 %; 1/42), and CC8/ST8-MRSA IV/t008 (2.4 %; 1/42) clonal lineages. Additionally, TST-positive strains belonged to CC8/ST239-MRSA III/t030 (66.6 %; 2/3) and CC8/ST239-MRSA III/t037 (33.4 %; 1/3). Isolates harboring *eta* gene belonged to the CC8/ST585-MRSA III/t713 (42.8 %; 3/7), CC30/ST30-MSSA/t318 (28.6 %; 2/7), and CC22/ST22-MSSA/t1869 (28.6 %; 2/7) lineages. Table 3 details the distribution of clones among the 60 *S. aureus* strains based on SCC*mec*, *spa,* and MLST typing.

## 4 Discussion

Previous studies have not specifically reported the occurrence rate of *S. aureus* involved in OE cases in Iran [3]. However, the general incidence of OE in the country varies between 12.4 % and 63.46 %. In our study, the prevalence of *S. aureus* isolates in OE patients was found to be 33.7 %. Notably, 15 % of these *S. aureus* isolates were resistant to fusidic acid. In a recently conducted meta-analysis by Hajikhani et al., a low prevalence rate of fusidic acid resistance in *S. aureus* isolates was reported (0.5 %) [14]. In line with our findings, they also noted a higher prevalence rate of fusidic acid resistance in *S. aureus* isolates from Asia (5.6 %) in comparison to other continents, which is potentially attributed to unrestricted and unscheduled administration of fusidic acid, a lack of proper alternatives, varied approaches towards antimicrobial management, and the dissemination of specific fusidic acid-resistant clones. The emergence of VRSA poses a significant challenge to controlling staphylococcal infections [15]. It is noteworthy that in our study, one isolate (1.7 %) displayed decreased susceptibility to vancomycin and carried *vanA* gene. A 2020 meta-analysis depicted an upward global trend in VRSA incidence. Shariati et al. reported a two-fold increase in VRSA cases after 2010 compared to prior years. Meanwhile, Asian countries, particularly Iran and India, have recorded the highest incidence rates of VRSA (67 %). This alarming trend may be associated with the easy availability of antibiotics, the scarcity of appropriate alternatives to vancomycin, inadequate monitoring of drug resistance patterns, and diverse antimicrobial treatment practices [16].

Mupirocin usage plays a crucial role in controlling the dissemination of *S. aureus* isolates in communities and healthcare settings, as well as in preventing severe infections [17,18]. In our study, the data showed that 13.3 % of MRSA isolates were resistant to mupirocin, all exhibiting the HLMUPR phenotype. The incidence rate of HLMUPR-MRSA in this study was reported to be higher than that of France (0.8 %), Canada (4.3 %), and China (7 %) [[18], [19], [20]]. Overall, the underlying causes for this increased rate of mupirocin resistance are not well understood but may be related to inadequate regulation of antibiotic use, flawed policies, and the overuse of this antibiotic. Therefore, it is essential to determine the mupirocin susceptibility of isolates before initiating mupirocin therapy.

In the present research, the 60 *S. aureus* isolates belonged to twelve particular *spa* types and were mostly clustered into the four main CCs (CC30, CC22, CC8, and CC1). The majority of the detected CCs have been frequently reported in previous studies as common in *S. aureus* strains isolated from ear infections [21,22]. Our observations about clonal lineages indicated genetic diversity among the strains under study. CC30/ST30 isolates were identified in both MRSA and MSSA strains. The most prevalent type was CC30/ST30-MRSA IV/t019-PVL+ (41.7 %), also known as the pandemic Southwest Pacific (SWP) clone or USA1100. This PVL-producing clone has been previously reported in various regions, including Qatar (8 %), the UAE (11 %), Saudi Arabia (12.2 %), Kuwait (2.5 %), and some European countries [23,24]. The CC30/ST30-MRSA IV/t605-PVL^+^ was the second most common, characterized by a 100 % prevalence of the cMLSB phenotype. This finding aligns with prior research conducted by Boswihi et al. in Kuwait, who indicated CC30/ST30-MRSA IV/t605 as the newly detected genotype in MRSA strains isolated from hospitalized individuals [24]. Our study aligns with previous findings by Goudarzi et al., who reported the emergence of CC30/ST30-MRSA IV/t605-PVL+ with the iMLSB phenotype in 1.6 % of tested isolates [25]. In our collection of MSSA isolates, CC/ST30-MSSA/t021 and CC30/ST30-MSSA/t318 represented 5 % and 10 % of isolates, respectively. This supports the earlier findings of Tayebi et al. in Iran, who indicated CC30/ST30-MSSA-t021 and CC30/ST30-MSSA/t318 as the third most detected genotypes in MSSA strains isolated from hospitalized patients, accounting for 9.4 % and 1.2 %, respectively [26]. The CC30/ST30-MSSA/t318 clone was previously reported in Taiwan, the UAE, Iran, Europe, Saudi Arabia, and Egypt [23]. Contrary to our findings, Goudarzi et al. reported a higher frequency of the CC30/ST30-MSSA/t318 clone in healthcare settings in Iran, representing 15 % of the MSSA isolates. Their study also showed a significant increasing trend for this clone, from 8 % in 2013 to 18.4 % in 2018 [25]. These differences may reflect differences in study design, sampling methods, laboratory techniques, sample size, population diversity, and temporal trends, suggesting the prevalence of this clone may be dynamic. However, the results indicate the potential risk of these isolates in nosocomial infections, which merits more attention. Undoubtedly, further studies with a larger sample size and from different regions of the country are necessary to reach a comprehensive conclusion. Additionally, a study by Wurster et al. in 2018 reported CC30/ST30 as the second predominant *S. aureus* clone isolated from ear infections, accounting for 19.4 % of cases [21]. Surprisingly, our data exhibited that all tested isolates had the ability to form biofilms at different intensities, confirming the earlier findings of Chamon et al. from Brazil, which indicated a high rate of biofilm formation among CC/ST30 strains [27].

The current study highlighted the emergence of CC22/ST22, which represents 13.3 % of the *S. aureus* isolates associated with otitis externa (OE) infections. This group comprises three clones: CC22/ST22-MSSA/t005 at 8.3 %; CC22/ST22-MSSA/t1869 at 3.3 %; and CC22/ST22-MRSA IV/t790 at 1.7 %, marking it as the second predominant with a notable carriage of *pvl* (75 %). This observation aligns with a UK-based study where CC22/ST22-*spa* t005 was reported to be the most common clone (47.4 %) identified among PVL-MSSA strains [28]. The present result is consistent with an Irish study conducted between 2002 and 2011, which reported a low prevalence of CC22/ST22-MSSA/t005 and CC22/ST22-MSSA/t1869 strains [29]. In contrast with earlier results that identified CC22/ST22-MRSA IV/t790 as the most frequent type among *S. aureus* from pathological specimens, our data displayed a relatively low prevalence (1.7 %) of this *spa* type in *S. aureus* related to OE. Similar prevalence levels have been reported in regional studies, including those by Boswihi et al. [30] in Kuwait and Goudarzi et al. [25] in Iran.

Our findings further displayed a relatively low frequency of CC8/ST8, corresponding to *spa* type t008, CC8/ST239, which corresponded to two *spa* types t030, t037, and CC8/ST585, corresponding to *spa* type t713, as the third prevailing clone group among the isolates. The CC8/ST8-MRSA IV/t008 strain carrying *pvl* encoding genes akin to the USA300 has been previously reported in Australia, China, Iran, Japan, Kuwait, Spain, Switzerland, and the UAE [23,24]. According to the published data, multi-drug resistance, especially resistance to vancomycin and mupirocin, among USA300 strains is increasing globally. In alignment with present research, a 2012 study by Havaei et al. reported that VISA strains belonged to CC8/ST8-*spa* t008 [31].

Furthermore, we found that all types of CC8/ST239-MRSA III/t030 and CC8/ST239-MRSA III/t037 were resistant to fusidic acid. The outcomes of this study are in agreement with former research by Goudarzi et al. in Iran, which reported CC8/ST8-MRSA III/t030 strain as a common fusidic acid-resistant genotype [32]. Moreover, Yu et al. in China reported that the molecular type CC8/ST239-MRSA III/t030 carrying the *fusB* gene was observed in 7.1 % of the MRSA isolates [33]. Chen et al. in Taiwan further reported two molecular types, CC8/ST239-MRSA III/t037 and CC5/ST5-MRSA-II/t002, as the predominant genotypes in fusidic acid-resistant MRSA strains isolated from hospitalized individuals, accounting for 62 % and 29 %, respectively [34]. Our observations illustrated molecular type CC1/ST772-MRSA IV/t10795 [PVL+] in 8.3 % of all strains, which resembles the Bengal Bay clone. This genotype has been previously reported in Australia, Bangladesh, India, Ireland, Italy, the KSA, Kuwait, Malaysia, Nepal, New Zealand, the UAE, and the UK [23,24,29].

We also detected that the majority of CC30/ST30 strains (65 %) and lower percentages of CC1/ST772 (40 %), CC8/ST239, ST8 (28.6 %), and CC/ST22 (12.5 %) showed biofilm formation at a strong level. Our analysis also supports the data published from the Netherlands by Croes et al., which reported a high rate of occurrence of CC/ST8, ST11, CC/ST1, CC/ST5, CC/ST22, CC30/ST36, and CC/ST45 among strong biofilm-forming strains. They also indicated a high capacity of CC8/ST239 strains in biofilm formation with strong intensity [35]. Similarly, a study conducted by Atshan et al. reported that approximately more than half of the CC8/ST239 isolates and less than one-fifth of CC1/ST1, CC22/ST22, and CC7/ST7 were confirmed as the most frequent biofilm producers in Malaysia [36]. The variation in the potential of biofilm formation among these clonal lineages could be attributed to a distinctive assemblage of surface-associated and regulatory genes [35].

One of the limitations of the current study is its small sample size. Additionally, the lack of funds to perform whole genome sequencing techniques is also a limitation of this study. However, it is important to note that while many isolates may have genetic and epidemiological connections, it is crucial to obtain a larger number of clinical isolates to accurately assess the resistance rate across various epidemiological backgrounds.

## 5 Conclusion

This study provides knowledge about the local molecular epidemiology of *S. aureus* related to OE infections in Iran. Our findings confirmed the dissemination of specific types among *S. aureus* strains causing ear infections. Alarmingly, the high prevalence of CC30/ST30, identified as a predominant clone in OE cases, raises concerns about its potential to trigger an epidemic in Iran in the future. Further research involving larger populations of OE cases is essential to develop a comprehensive molecular epidemiological map.

## Data availability statement

Data included in article/supp. Material/referenced in the article:

## CRediT authorship contribution statement

**Zahra Rahmani:** Methodology, Formal analysis, Data curation. **Sareh Sadat Hosseini:** Writing – review & editing, Writing – original draft, Methodology, Formal analysis. **Parmida Bagheri:** Writing – original draft, Investigation, Data curation. **Masoud Dadashi:** Project administration, Methodology, Investigation, Formal analysis. **Mehrdad Haghighi:** Project administration, Methodology. **Mehdi Goudarzi:** Writing – review & editing, Writing – original draft, Validation, Supervision, Resources, Project administration, Methodology, Funding acquisition, Conceptualization.

## Declaration of competing interest

The authors declare the following financial interests/personal relationships which may be considered as potential competing interests:Mehdi Goudarzi reports financial support was provided by 10.13039/501100005851Shahid Beheshti University of Medical Sciences School of Medicine. If there are other authors, they declare that they have no known competing financial interests or personal relationships that could have appeared to influence the work reported in this paper.
