# Supplementary material for: Phenotypic and genotypic characterization of Staphylococcus aureus strains isolated from otitis externa: Emergence of CC30-spa t019-SCCmec IV carrying PVL as major genotype
Source: Heliyon. 2024 May 28;10(11):e32002. doi: 10.1016/j.heliyon.2024.e32002 (PMC11168311; doi:10.1016/j.heliyon.2024.e32002)
Supplement: Multimedia component 1 [file mmc1.docx]

Table S1. Oligonucleotide primers used in this study

| Target | primer | Primer sequence (5´ 3´) | Product size (bp) | Reference |
| --- | --- | --- | --- | --- |
| *nucA* | F | GCGATTGATGGTGATACGGTT | 270 | Pinto *et al.* |
|  | R | AGCCAAGCCTTGACGAACTAAAGC |  |  |
| *mecA* | F | AGAAGATGGTATGTGGAAGTTAG | 583 | Azimian *et al.* |
|  | R | ATGTATGTGCGATTGTATTGC |  |  |
| *luk-PV* | F | TTCACTATTTGTAAAAGTGTCAGACCCACTT | 180 | Goudarzi *et al.* |
|  | R | ACTAATGAATTTTTTTATCGTAAGCCCTT |  |  |
| *tst* | F | TTATCGTAAGCCCTTTGTTG | 398 | Azimian *et al.* |
|  | R | TAAAGGTAGTTCTATTGGAGTAGG |  |  |
| *eta* | F | GCAGGTGTTGATTTAGCATT | 93 | Stegger *et al.* |
|  | R | AGATGTCCCTATTTTTGCTG |  |  |
| *etb* | F | ACAAGCAAAAGAATACAGCG | 226 | Koosha *et al.* |
|  | R | GTTTTTGGCTGCTTCTCTTG |  |  |
| *VanA* | F | GGCAAGTCAGGTGAAGATG | 713 | Azimian *et al.* |
|  | R | ATCAAGCGGTCAATCAGTTC |  |  |
| *spa* | F | AGACGATCCTTCGGTGAGC | variable | Harmsen *et al.* |
|  | R | GCTTTTGCAATGTCATTTACTG |  |  |
| SCC*mec* | Fβ | ATTGCCTTGATAATAGCCYTCT | 937 | Boye *et al*. |
|  | R α3 | TAAAGGCATCAATGCACAAACACT |  |  |
|  | F ccrC | CGTCTATTACAAGATGTTAAGGATAAT | 518 |  |
|  | R ccrC | CCTTTATAGACTGGATTATTCAAAATAT |  |  |
|  | F 1272 | GCCACTCATAACATATGGAA | 415 |  |
|  | R 1272 | CATCCGAGTGAAACCCAAA |  |  |
|  | F 5R*mec*A | TATACCAAACCCGACAACTAC | 359 |  |
|  | R 5R431 | CGGCTACAGTGATAACATCC |  |  |
|  | F | ATCATTAGGTAAAATGTCTGGACATGATCCA | 433 |  |
|  | R | GCATCAAGTGTATTGGATAGCAAAAGC |  |  |

Table S2: Primer sequences and PCR conditions for MLST.

| **Target** | **Product** | **Primer sequences (5´→3´)** | **Annealing temperature** | **Amplicon size (bp)** | **Reference** |
| --- | --- | --- | --- | --- | --- |
| *arc* | Carbamate kinase | F: TTGATTCACCAGCGCGTATTGTC | 55°C | 456 | https://pubmlst.org/organisms/staphylococcus-aureus/primers |
|  |  | R: AGGTATCTGCTTCAATCAGCG |  |  |  |
| *aro* | Shikimate dehydrogenase | F: ATCGGAAATCCTATTTCACATTC | 55°C | 456 |  |
|  |  | R: GGTGTTGTATTAATAACGATATC |  |  |  |
| *glp* | Glycerol kinase | F: CTAGGAACTGCAATCTTAATCC | 55°C | 465 |  |
|  |  | R: TGGTAAAATCGCATGTCCAATTC |  |  |  |
| *gmk* | Guanylate kinase | F: ATCGTTTTATCGGGACCATC | 55°C | 429 |  |
|  |  | R: TCATTAACTACAACGTAATCGTA |  |  |  |
| *pta* | Phosphate acetyltransferase | F: GTTAAAATCGTATTACCTGAAGG | 55°C | 474 |  |
|  |  | R: GACCCTTTTGTTGAAAAGCTTAA |  |  |  |
| *tpi* | Triosephosphate isomerase | F: TCGTTCATTCTGAACGTCGTGAA | 55°C | 402 |  |
|  |  | R: TTTGCACCTTCTAACAATTGTAC |  |  |  |
| *yqi* | Acetyle coenzyme A acetyltransferase | F: CAGCATACAGGACACCTATTGGC | 55°C | 516 |  |
|  |  | R: CGTTGAGGAATCGATACTGGAAC |  |  |  |


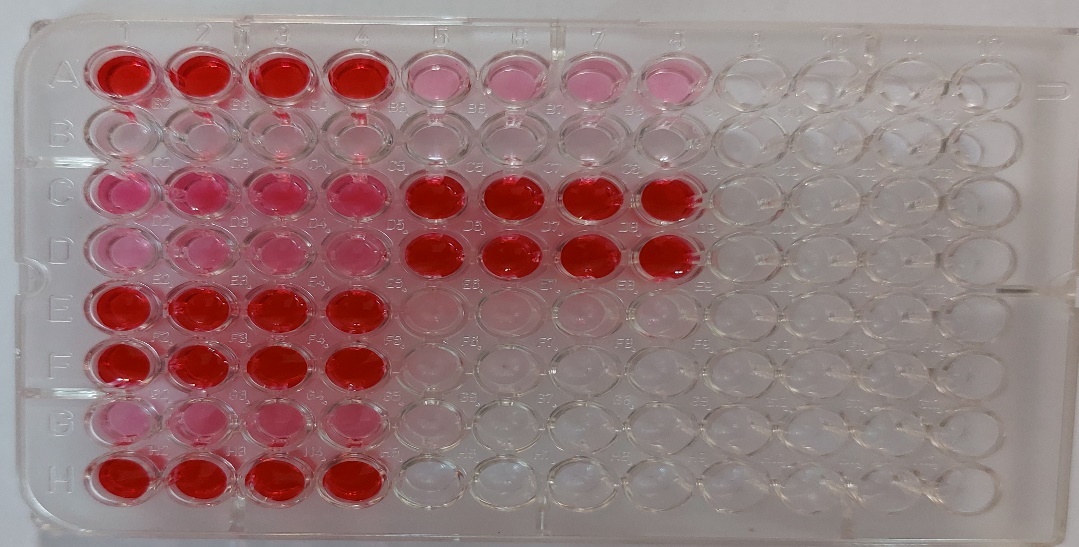


Figure S1. Results of microtiter plate (MTP) assay. Column A 1–4 represent positive control (*S. epidermidis* ATCC 35984 strain), B 1-4 represent negative control (sterile tryptic soy broth medium), C1-4 represent moderate biofilm formation, D 1-4 weak biofilm formation, and E 1-4 represent the strong biofilm formation.


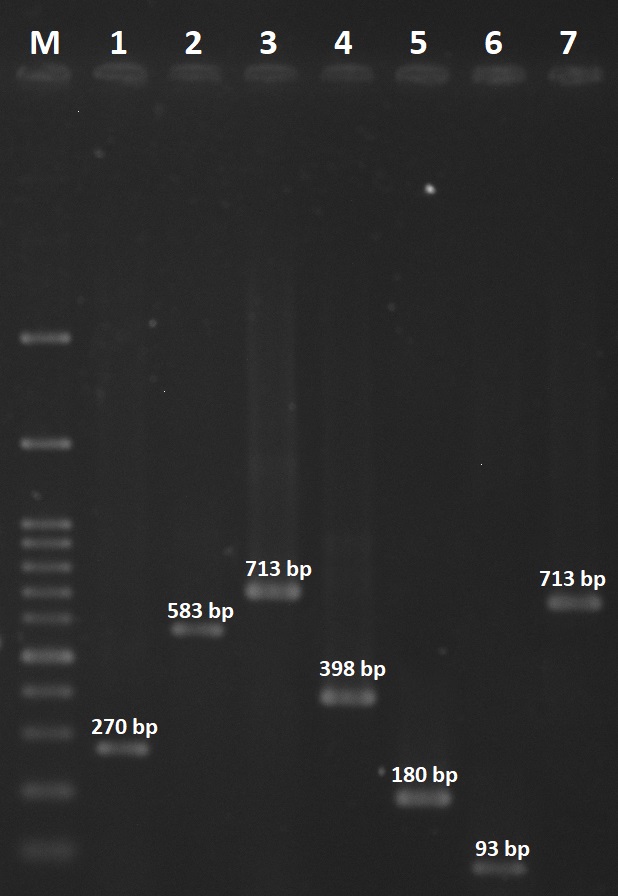


**Figure S2**. PCR showing positive amplification of 270 base fragments specific for *nucA* of *S. aureus,* 583 base pair fragments specific for the *mecA* gene, 713 base pair fragments specific for the *vanA* gene, 398 base pair fragments specific for the *tst* gene, 180 base pair fragments specific for the *pvl* gene, 93 base pair fragments specific for the *eta* gene, and 713 base pair fragments specific for the *vanA* gene.


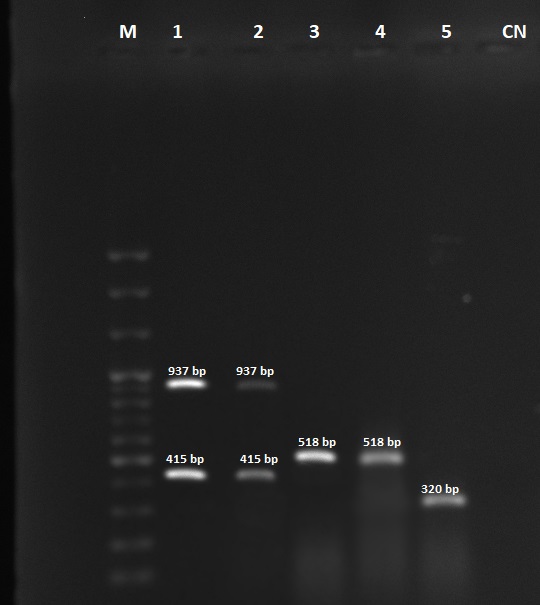


**Figure S3**. SCC*mec* typing of *S. aureus* isolates. Lane M, 100-bp DNA Ladder (Fermentas, Vilnius, Lithuania); Lane 1 SCC*mec* type IV positive control (937 and 415 bp), Lane 2 SCC*mec* type IV (937 and 415 bp), Lane 3 SCC*mec* type III positive control (518 bp), Lane 4 SCC*mec* type III (518 bp), Lane 5 *spa* gene (320 bp), and CN control negative.


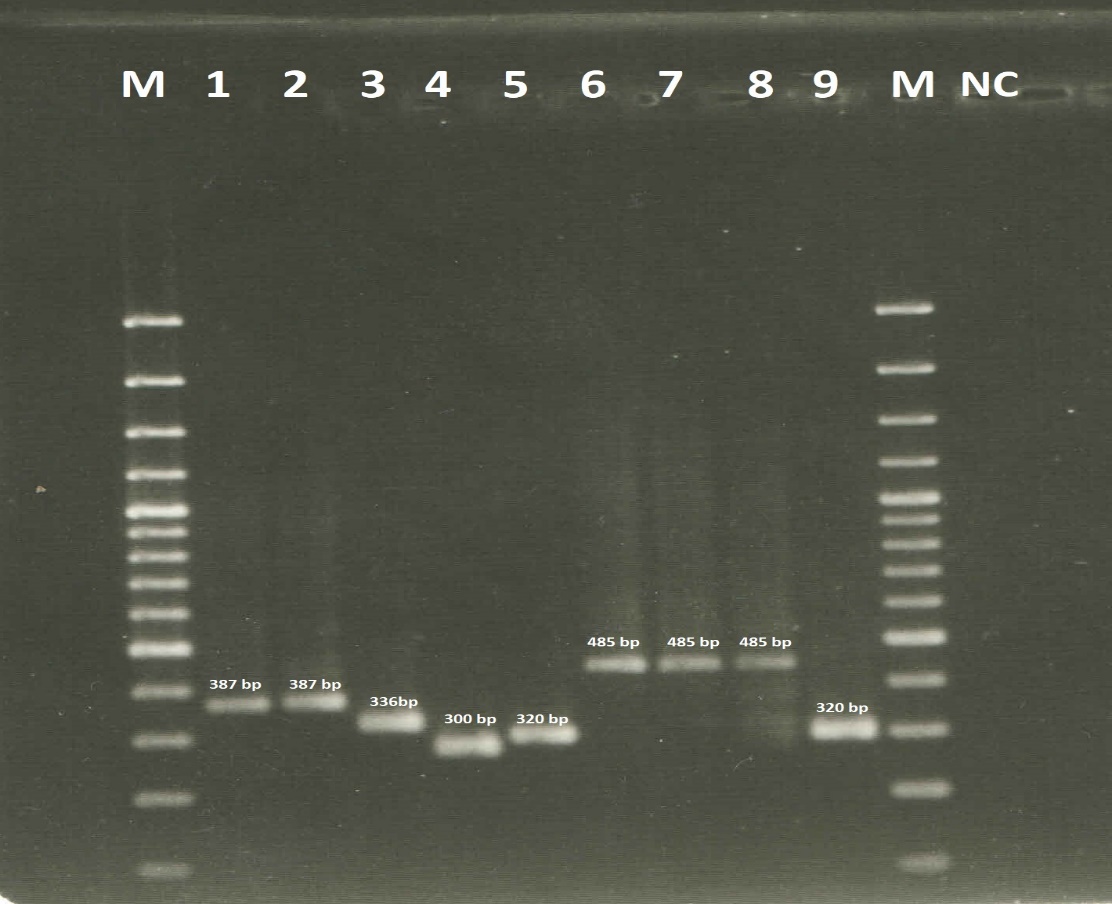


**Figure S4**. Results of *spa* PCR Lane M, 100-bp DNA Ladder (Fermentas, Vilnius, Lithuania); Lane 1 positive control (387 bp), Lane 2-9 variable size of spa gene (387 bp, 336 bp, 300 bp, 320 bp, and 485 bp), and Lane NC negative control.

Table S3. *spa* gene tandem repeats, *spa* types, and sequence of *S. aureus* isolates

| No of isolates (%) | Kreiswirth ID | *spa* type | Repeat | FASTA Sequence |
| --- | --- | --- | --- | --- |
| 25 (41.7) | XKAKAOMQ | t019 | 08-16-02-16-02-25-17-24 | ˃019 GAGGAAGACAACAACAAGCCTGGTAAAGAAGACGGCAACAAACCTGGTAAAGAAGACAACAAAAAACCTGGCAAAGAAGACGGCAACAAACCTGGTAAAGAAGACAACAAAAAACCTGGCAAAGAAGATGGCAACAAACCTGGTAAAGAAGACGGCAACAAGCCTGGTAAAGAAGATGGCAACAAGCCTGGT |
| 6 (10) | UJ | t605 | 07-23 | ˃605 GAGGAAGACAACAACAAACCTGGTAAAGAAGACGGCAACAAACCTGGC |
| 6 (10) | WGKKAKAOMQ | t318 | 15-12-16-16-02-16-02-25-17-24 | ˃318 GAGGAAGACAACAACAAGCCTGGCAAAGAAGACAACAACAAGCCTGGTAAAGAAGACGGCAACAAACCTGGTAAAGAAGACGGCAACAAACCTGGTAAAGAAGACAACAAAAAACCTGGCAAAGAAGACGGCAACAAACCTGGTAAAGAAGACAACAAAAAACCTGGCAAAGAAGATGGCAACAAACCTGGTAAAGAAGACGGCAACAAGCCTGGTAAAGAAGATGGCAACAAGCCTGGT |
| 5 (8.3) | TJEJNCMOMOKR | t005 | 26-23-13-23-31-05-17-25-17-25-16-28 | ˃005 GAGGAAGACAACAAAAAACCTGGTAAAGAAGACGGCAACAAACCTGGCAAAGAAGACAACAACAAACCTGGTAAAGAAGACGGCAACAAACCTGGCAAAGAAGATGGCAACAAACCTGGCAAAGAAGACAACAAAAAGCCTGGCAAAGAAGACGGCAACAAGCCTGGTAAAGAAGATGGCAACAAACCTGGTAAAGAAGACGGCAACAAGCCTGGTAAAGAAGATGGCAACAAACCTGGTAAAGAAGACGGCAACAAACCTGGTAAAGAAGATGGTAACAAACCTGGC |
| 5 (8.3) | TJEFMBBPE | t10795 | 26-23-13-21-17-34-34-33-13 | ˃10795 GAGGAAGACAACAAAAAACCTGGTAAAGAAGACGGCAACAAACCTGGCAAAGAAGACAACAACAAACCTGGTAAAGAAGACAACAACAAGCCTGGCAAAGAAGACGGCAACAAGCCTGGTAAAGAAGACAACAAAAAACCTGGTAAAGAAGACAACAAAAAACCTGGTAAAGAAGATGGCAACAAGCCTGGCAAAGAAGACAACAACAAACCTGGT |
| 3 (5) | WGKAKAOMQ | t021 | 15-12-16-02-16-02-25-17-24 | ˃021 GAGGAAGACAACAACAAGCCTGGCAAAGAAGACAACAACAAGCCTGGTAAAGAAGACGGCAACAAACCTGGTAAAGAAGACAACAAAAAACCTGGCAAAGAAGACGGCAACAAACCTGGTAAAGAAGACAACAAAAAACCTGGCAAAGAAGATGGCAACAAACCTGGTAAAGAAGACGGCAACAAGCCTGGTAAAGAAGATGGCAACAAGCCTGGT |
| 3 (5) | XKBMM | t713 | 08-16-34-17-17 | ˃713 GAGGAAGACAACAACAAGCCTGGTAAAGAAGACGGCAACAAACCTGGTAAAGAAGACAACAAAAAACCTGGTAAAGAAGACGGCAACAAGCCTGGTAAAGAAGACGGCAACAAGCCTGGT |
| 2 (3.3) | TJBJNCMOMOKR | t1869 | 26-23-34-23-31-05-17-25-17-25-16-28 | ˃1869 GAGGAAGACAACAAAAAACCTGGTAAAGAAGACGGCAACAAACCTGGCAAAGAAGACAACAAAAAACCTGGTAAAGAAGACGGCAACAAACCTGGCAAAGAAGATGGCAACAAACCTGGCAAAGAAGACAACAAAAAGCCTGGCAAAGAAGACGGCAACAAGCCTGGTAAAGAAGATGGCAACAAACCTGGTAAAGAAGACGGCAACAAGCCTGGTAAAGAAGATGGCAACAAACCTGGTAAAGAAGACGGCAACAAACCTGGTAAAGAAGATGGTAACAAACCTGGC |
| 2 (3.3) | WGKAQQ | t030 | 15-12-16-02-24-24 | ˃030 GAGGAAGACAACAACAAGCCTGGCAAAGAAGACAACAACAAGCCTGGTAAAGAAGACGGCAACAAACCTGGTAAAGAAGACAACAAAAAACCTGGCAAAGAAGATGGCAACAAGCCTGGTAAAGAAGATGGCAACAAGCCTGGT |
| 1 (1.7) | TJEJNFMOMOKR | t790 | 26-23-13-23-31-29-17-25-17-25-16-28 | ˃790 GAGGAAGACAACAAAAAACCTGGTAAAGAAGACGGCAACAAACCTGGCAAAGAAGACAACAACAAACCTGGTAAAGAAGACGGCAACAAACCTGGCAAAGAAGATGGCAACAAACCTGGCAAAGAAGACAACAAAAAGCCTAGCAAAGAAGACGGCAACAAGCCTGGTAAAGAAGATGGCAACAAACCTGGTAAAGAAGACGGCAACAAGCCTGGTAAAGAAGATGGCAACAAACCTGGTAAAGAAGACGGCAACAAACCTGGTAAAGAAGATGGTAACAAACCTGGC |
| 1 (1.7) | WGKAOMQ | t037 | 15-12-16-02-25-17-24 | ˃037 GAGGAAGACAACAACAAGCCTGGCAAAGAAGACAACAACAAGCCTGGTAAAGAAGACGGCAACAAACCTGGTAAAGAAGACAACAAAAAACCTGGCAAAGAAGATGGCAACAAACCTGGTAAAGAAGACGGCAACAAGCCTGGTAAAGAAGATGGCAACAAGCCTGGT |
| 1 (1.7) | YHGFMBQBLO | t008 | 11-19-12-21-17-34-24-34-22-25 | ˃008 GAGGAAGACAATAACAAGCCTGGCAAAGAAGACAATAACAAGCCTGGCAAAGAAGACAACAACAAGCCTGGTAAAGAAGACAACAACAAGCCTGGCAAAGAAGACGGCAACAAGCCTGGTAAAGAAGACAACAAAAAACCTGGTAAAGAAGATGGCAACAAGCCTGGTAAAGAAGACAACAAAAAACCTGGTAAAGAAGACGGCAACAAGCCTGGC AAAGAAGATGGCAACAAACCTGGT |

| **ST** | **arcC** | **aroE** | **glpF** | **gmk** | **pta** | **tpi** | **yqiL** |
| --- | --- | --- | --- | --- | --- | --- | --- |
| 30 | 2 | 2 | 2 | 2 | 6 | 3 | 2 |
| 22 | 7 | 6 | 1 | 5 | 8 | 8 | 6 |
| 585 | 2 | 1 | 1 | 1 | 4 | 4 | 3 |
| 239 | 2 | 3 | 1 | 1 | 4 | 4 | 3 |
| 8 | 3 | 3 | 1 | 1 | 4 | 4 | 3 |
| 772 | 1 | 1 | 1 | 1 | 22 | 1 | 1 |

Table S4. Allelic profile of different ST types of *S. aureus* isolates

Table 5. Nucleotide sequence among alleles of 7 MLST housekeeping genes of *S. aureus* isolates

| **Sequence type** | **Nucleotide sequence** |
| --- | --- |
| **30** | **>arcC_2**  TTATTAATCCAACAAGCTAAATCGAACAGTGACACAACGCCGGCAATGCCATTGGATACTTGTGGTGCAATGTCACAAGGTATGATAGGCTATTGGTTGGAAACTGAAATCAATCGCATTTTAACTGAAATGAATAGTGATAGAACTGTAGGCACAATCGTAACACGTGTGGAAGTAGATAAAGATGATCCACGATTTGATAACCCAACTAAACCAATTGGTCCTTTTTATACGAAAGAAGAAGTTGAAGAATTACAAAAAGAACAGCCAGGCTCAGTCTTTAAAGAAGATGCAGGACGTGGTTATAGAAAAGTAGTTGCGTCACCACTACCTCAATCTATACTAGAACACCAGTTAATTCGAACTTTAGCAGACGGTAAAAATATTGTCATTGCATGCGGTGGTGGCGGTATTCCAGTTATAAAAAAAGAAAATACCTATGAAGGTGTTGAAGCG  **>aroE_2**  AATTTTAATTCTTTAGGATTAGATGATACTTATGAAGCTTTAAATATTCCAATTGAAGATTTTCATTTAATTAAAGAAATTATTTCAAAAAAAGAATTAGATGGCTTTAATATCACAATTCCTCATAAAGAGCGTATCATACCGTATTTAGATCATGTTGATGAACAAGCGATTAATGCAGGTGCAGTTAACACTGTTTTGATAAAAGATGGCAAGTGGATAGGGTATAATACAGATGGTATTGGTTATGTTAAAGGATTGCACAGCGTTTATCCAGATTTAGAAAATGCATACATTTTAATTTTGGGAGCAGGTGGTGCAAGTAAAGGTATTGCTTATGAATTAGCAAAATTTGTAAAGCCCAAATTAACTGTTGCGAATAGAACGATGGCTCGTTTTGAATCTTGGAATTTAAATATAAACCAAATTTCATTGGCAGATGCTGAAAAGTATTTA  **>glpF_2**  GGTGCTGATTGGATTGTCATCACAGCTGGATGGGGATTAGCGGTTACAATGGGTGTGTATGCTGTTGGTCAATTCTCAGGTGCACATTTAAACCCAGCGGTGTCTTTAGCTCTTGCATTAGACGGAAGTTTTGATTGGTCATTAGTTCCTGGTTATATTGTTGCTCAAATGTTAGGTGCAATTGTCGGAGCAACAATTGTATGGTTAATGTACTTGCCACATTGGAAAGCGACAGAAGAAGCTGGCGCGAAATTAGGTGTTTTCTCTACAGCACCGGCTATTAAGAATTACTTTGCCAACTTTTTAAGTGAAATTATCGGAACAATGGCATTAACTTTAGGTATTTTATTTATCGGTGTAAACAAAATTGCTGATGGTTTAAATCCTTTAATTGTCGGAGCATTAATTGTTGCAATCGGATTAAGTTTAGGCGGTGCTACTGGTTATGCAATCAACCCAGCACGT  **>gmk_2**  CGAATATTTGAAGATCCAAGTACATCATATAAGTATTCTATTTCAATGACAACACGTCAAATGCGTGAAGGTGAAGTTGATGGCGTAGATTACTTTTTTAAAACTAGGGATGCGTTTGAAGCTTTAATTAAAGATGACCAATTTATAGAATATGCTGAATATGTAGGCAACTATTATGGTACACCAGTTCAATATGTTAAAGATACAATGGACGAAGGTCATGATGTATTTTTAGAAATTGAAGTAGAAGGTGCAAAGCAAGTTAGAAAGAAATTTCCAGATGCGTTATTTATTTTCTTAGCACCTCCAAGTTTAGATCACTTGAGAGAGCGATTAGTAGGTAGAGGAACAGAATCTGATGAGAAAATACAAAGTCGTATTAACGAAGCACGTAAAGAAGTCGAAATGATGAATTTA  **>pta_6**  GCAACACAATTACAAGCAACAGATTATGTTACACCAATCGTGTTAGGTGATGAGACTAAGGTTCAATCTTTAGCGCAAAAACTTAATCTTGATATTTCTAATATTGAATTAATTAATCCTGCGACAAGTGAATTGAAAGCTGAATTAGTTCAATCATTTGTTGAACGACGTAAAGGTAAAGCGACTGAAGAACAAGCACAAGAATTATTAAACAATGTGAACTACTTCGGTACAATGCTTGTTTATGCTGGTAAAGCAGATGGTTTAGTTAGTGGTGCAGCACATTCAACAGGCGACACTGTGCGTCCAGCTTTACAAATCATCAAAACGAAACCAGGTGTATCAAGAACATCAGGTATCTTCTTTATGATTAAAGGTGATGAACAGTACATCTTTGGTGATTGTGCAATCAATCCAGAACTTGATTCACAAGGACTTGCAGAAATTGCAGTAGAAAGTGCAAAATCAGCATTA  **>tpi_3**  CACGAAACAGATGAAGAAATTAACAAAAAAGCGCACGCTATTTTCAAACATGGAATGACTCCAATTATTTGTGTTGGTGAAACAGACGAAGAGCGTGAAAGTGGTAAAGCTAACGATGTTGTAGGTGAGCAAGTTAAGAAAGCTGTTGCAGGTTTATCTGAAGATCAACTTAAATCAGTTGTAATTGCTTATGAACCAATCTGGGCAATCGGAACTGGTAAATCATCAACATCTGAAGATGCGAATGAAATGTGTGCATTTGTACGTCAAACTATTGCTGACTTATCAAGCAAAGAAGTATCAGAAGCAACTCGTATTCAATATGGTGGTAGTGTTAAACCTAACAACATTAAAGAATACATGGCACAAACTGATATTGATGGGGCATTAGTAGGTGGCGCA  **>yqiL_2**  GCGTTTAAAGACGTGCCAGCCTATGATTTAGGTGCGACTTTAATAGAACATATTATTAAAGAGACGGGTTTGAATCCAAGTGAGATTAATGAAGTCATCATCGGTAACGTACTACAAGCAGGACAAGGACAAAATCCAGCACGAATTGCTGCTATGAAAGGTGGCTTGCCAGAAACAGTACCTGCATTTACAGTGAATAAAGTATGTGGTTCTGGGTTAAAGTCGATTCAATTAGCATATCAATCTATTGTGACTGGTGAAAATGACATCGTGCTAGCTGGCGGTATGGAGAATATGTCTCAATCACCAATGCTTGTCAACAACAGTCGCTTTGGTTTTAAAATGGGACATCAATCAATGGTTGATAGCATGGTATATGATGGTTTAACAGATGTATTTAATCAATATCATATGGGTATTACTGCTGAAAATTTAGTAGAGCAATATGGTATTTCAAGAGAAGAACAAGATACATTTGCTGTAAACTCACAACAAAAAGCAGTACGTGCACAGCAA |
| **22** | **>arcC_7**  TTATTAATCCAACAAGCTAAATCGAACAGTGACACAACGCCGGCAATGCCATTGGATACTTGTGGTGCAATGTCACAGGGTATGATAGGCTATTGGTTGGAAACTGAAATCAATCGCATTTTAACTGAAATGAATAGTGATAGAACTGTAGGCACAATCGTTACACGTGTGGAAGTAGATAAAGATGATCCACGATTCAATAACCCAACCAAACCAATTGGTCCTTTTTATACGAAAGAAGAAGTTGAAGAATTACAAAAAGAACAGCCAGACTCAGTCTTTAAAGAAGATGCAGGACGTGGTTATAGAAAAGTTGTTGCGTCACCACTACCTCAATCTATACTAGAACACCAGTTAATTCGAACTTTAGCAGACGGTAAAAATATTGTCATTGCATGCGGTGGTGGCGGTATTCCAGTTATAAAAAAAGAAAATACCTATGAAGGTGTTGAAGCG  **>aroE_6**  AATTTTAATTCTTTAGGATTAGATGATACTTATGAAGCTTTAAATATTCCAATTGAAGATTTTCATTTAATTAAAGAAATTATTTCGAAAAAAGAATTAGATGGCTTTAATATCACAATTCCTCATAAAGAGCGTATCATACCGTATTTAGATCATGTTGATGAACAAGCGATTAATGCAGGTGCAGTTAACACTGTTTTGATAAAAGATGGCAAGTGGATAGGGTATAATACAGATGGTATTGGTTATGTTAAAGGATTGCACAGCGTTTATCCAGATTTAGAAAATGCATACATTTTAATTTTGGGCGCAGGTGGTGCAAGTAAAGGTATTGCTTATGAATTAGCAAAATTTGTAAAGCCCAAATTAACTGTTGCGAATAGAACGATGGCTCGTTTTGAATCTTGGAATTTAAATATAAACCAAATTTCAATAGCAGATGCTGAAAAGTATTTA  **>glpF_1**  GGTGCTGATTGGATTGTCATCACAGCTGGATGGGGATTAGCGGTTACAATGGGTGTGTTTGCTGTCGGTCAATTCTCAGGTGCACATTTAAACCCAGCGGTGTCTTTAGCTCTTGCATTAGACGGAAGTTTTGATTGGTCATTAGTTCCTGGTTATATTGTTGCTCAAATGTTAGGTGCAATTGTCGGAGCAACAATTGTATGGTTAATGTACTTGCCACATTGGAAAGCGACAGAAGAAGCTGGCGCGAAATTAGGTGTTTTCTCTACAGCACCGGCTATTAAGAATTACTTTGCCAACTTTTTAAGTGAGATTATCGGAACAATGGCATTAACTTTAGGTATTTTATTTATCGGTGTAAACAAAATTGCCGATGGTTTAAATCCTTTAATTGTCGGAGCATTAATTGTTGCAATCGGATTAAGTTTAGGCGGTGCTACTGGTTATGCAATCAACCCAGCACGT  **>gmk_5**  CGAATATTTGAAGATCCAAGTACATCATATAAGTATTCTATTTCAATGACAACACGTCAAATGCGTGAAGGTGAAGTTGATGGCGTAGATTACTTTTTTAAAACTAGGGATGCGTTTGAAGCTTTAATCAAAGATGACCAATTTATAGAATATGCTGAATATGTAGGCAACTATTATGGTACACCAGTTCAATATGTTAAAGATACAATGGACGAAGGTCATGATGTATTTTTAGAAATTGAAGTAGAAGGTGCAAAGCAAGTTAGAAAGAAATTTCCAGATGCATTATTTATTTTCTTAGCACCTCCAAGTTTAGATCACTTGAGAGAGCGATTAGTAGGTAGAGGAACAGAATCCGATGAGAAAATACAAAGTCGTATTAACGAAGCACGTAAAGAAGTCGAAATGATGAATTTA  **>pta_8**  GCAACACAATTACAAGCAACAGATTATGTTACACCAATCGTGTTAGGTGATGAGACTAAGGTTCAATCTTTAGCGCAAAAACTTAATCTTGATATTTCTAATATTGAATTAATTAATCCTGCGACAAGTGAATTTAAAGCTGAATTAGTCCAATCATTTGTTGAACGACGTAAAGGTAAAGCGACTGAAGAACAAGCGCAAGAATTATTAAACAATGTGAACTACTTCGGTACAATGCTTGTTTATGCTGGTAAAGCAGATGGTTTAGTTAGTGGTGCAGCACATTCAACAGGAGACACTGTGCGTCCAGCTTTACAAATCATCAAAACGAAACCAGGTGTATCAAGAACATCAGGTATCTTCTTTATGATTAAAGGTGATGAACAATACATCTTTGGTGATTGTGCAATCAATCCAGAACTTGATTCACAAGGACTTGCAGAAATTGCAGTAGAAAGTGCAAAATCAGCATTA  **>tpi_8**  CACGAAACAGATAAAGAAATTAACAAAAAAGCGCACGCTATTTTCAAACATGGAATGACTCCAATTATATGTGTTGGTGAAACAGACGAAGAGCGTGAAAGTGGTAAAGCTAACGATGTTGTAGGTGAGCAAGTTAAGAAAGCTGTTGCAGGTTTATCTGAAAATCAACTAAAATCAGTTGTAATTGCTTATGAACCAATCTGGGCAATCGGAACTGGTAAATCATCAACATCTGAAGATGCGAATGAAATGTGTGCATTTGTACGTCAAACTATTGCTGACTTATCAAGCAAAGAAGTATCAGAAGCAACTCGTATTCAATATGGTGGTAGTGTTAAACCTAACAACATTAAAGAATACATGGCACAAACTGATATTGATGGGGCATTAGTAGGTGGCGCA  **>yqiL_6**  GCGTTTAAAGACGTGCCAGCCTATGATTTAGGTGCGACTTTAATAGAACATATTATTAAAGAGACGGGTTTGAATCCAAGTGAGATTGATGAAGTTATCATCGGTAACGTACTACAAGCAGGACAAGGACAAAATCCAGCACGAATTGCTGCTATGAAAGGTGGCTTACCAGAGACAGTACCTGCATTTACAGTGAATAAAGTATGTGGTTCTGGGTTAAAGTCGATTCAATTAGCATATCAATCTATTGTGACTGGTGAAAATGACATCGTGCTAGCTGGCGGTATGGAGAATATGTCTCAATCACCAATGCTTGTCAACAACAGTCGCTTTGGTTTTAAAATGGGACATCAATCAATGGTTGATAGCATGGTATATGATGGTTTAACAGATGTATTTAATCAATATCATATGGGTATTACTGCTGAAAATTTAGTAGAGCAATATGGTATTTCAAGAGAAGAACAAGATACATTTGCTGTAAACTCACAACAAAAAGCAGTACGTGCACAGCAA |
| **585** | **>arcC_2**  TTATTAATCCAACAAGCTAAATCGAACAGTGACACAACGCCGGCAATGCCATTGGATACTTGTGGTGCAATGTCACAAGGTATGATAGGCTATTGGTTGGAAACTGAAATCAATCGCATTTTAACTGAAATGAATAGTGATAGAACTGTAGGCACAATCGTAACACGTGTGGAAGTAGATAAAGATGATCCACGATTTGATAACCCAACTAAACCAATTGGTCCTTTTTATACGAAAGAAGAAGTTGAAGAATTACAAAAAGAACAGCCAGGCTCAGTCTTTAAAGAAGATGCAGGACGTGGTTATAGAAAAGTAGTTGCGTCACCACTACCTCAATCTATACTAGAACACCAGTTAATTCGAACTTTAGCAGACGGTAAAAATATTGTCATTGCATGCGGTGGTGGCGGTATTCCAGTTATAAAAAAAGAAAATACCTATGAAGGTGTTGAAGCG  **>aroE_1**  AATTTTAATTCTTTAGGATTAGATGATACTTATGAAGCTTTAAATATTCCAATTGAAGATTTTCATTTAATTAAAGAAATTATTTCGAAAAAAGAATTAGATGGCTTTAATATCACAATTCCTCATAAAGAACGTATCATACCGTATTTAGATCATGTTGATGAACAAGCGATTAATGCAGGTGCAGTTAACACTGTTTTGATAAAAGATGACAAGTGGATAGGGTATAATACAGATGGTATTGGTTATGTTAAAGGATTGCACAGCGTTTATCCAGATTTAGAAAATGCATACATTTTAATTTTGGGCGCAGGTGGTGCAAGTAAAGGTATTGCTTATGAATTAGCAAAATTTGTAAAGCCCAAATTAACTGTTGCGAATAGAACGATGGCTCGTTTTGAATCTTGGAATTTAAATATAAACCAAATTTCATTAGCAGATGCTGAAAAGTATTTA  **>glpF_1**  GGTGCTGATTGGATTGTCATCACAGCTGGATGGGGATTAGCGGTTACAATGGGTGTGTTTGCTGTCGGTCAATTCTCAGGTGCACATTTAAACCCAGCGGTGTCTTTAGCTCTTGCATTAGACGGAAGTTTTGATTGGTCATTAGTTCCTGGTTATATTGTTGCTCAAATGTTAGGTGCAATTGTCGGAGCAACAATTGTATGGTTAATGTACTTGCCACATTGGAAAGCGACAGAAGAAGCTGGCGCGAAATTAGGTGTTTTCTCTACAGCACCGGCTATTAAGAATTACTTTGCCAACTTTTTAAGTGAGATTATCGGAACAATGGCATTAACTTTAGGTATTTTATTTATCGGTGTAAACAAAATTGCCGATGGTTTAAATCCTTTAATTGTCGGAGCATTAATTGTTGCAATCGGATTAAGTTTAGGCGGTGCTACTGGTTATGCAATCAACCCAGCACGT  **>gmk_1**  CGAATATTTGAAGATCCAAGTACATCATATAAGTATTCTATTTCAATGACAACACGTCAAATGCGTGAAGGTGAAGTTGATGGCGTAGATTACTTTTTTAAAACTAGGGATGCGTTTGAAGCTTTAATCAAAGATGACCAATTTATAGAATATGCTGAATATGTAGGCAACTATTATGGTACACCAGTTCAATATGTTAAAGATACAATGGACGAAGGTCATGATGTATTTTTAGAAATTGAAGTAGAAGGTGCAAAGCAAGTTAGAAAGAAATTTCCAGATGCGCTATTTATTTTCTTAGCACCTCCAAGTTTAGAACACTTGAGAGAGCGATTAGTAGGTAGAGGAACAGAATCTGATGAGAAAATACAAAGTCGTATTAACGAAGCGCGTAAAGAAGTTGAAATGATGAATTTA  **>pta_4**  GCAACACAATTACAAGCAACAGATTATGTTACACCAATCGTGTTAGGTGATGAGACTAAGGTTCAATCTTTAGCGCAAAAACTTGATCTTGATATTTCTAATATTGAATTAATTAATCCTGCGACAAGTGAATTGAAAGCTGAATTAGTTCAATCATTTGTTGAACGACGTAAAGGTAAAGCGACTGAAGAACAAGCACAAGAATTATTAAACAATGTGAACTACTTCGGTACAATGCTTGTTTATGCTGGTAAAGCAGATGGTTTAGTTAGTGGTGCAGCACATTCAACAGGCGACACTGTGCGTCCAGCTTTACAAATCATCAAAACGAAACCAGGTGTATCAAGAACATCAGGTATCTTCTTTATGATTAAAGGTGATGAACAATACATCTTTGGTGATTGTGCAATCAATCCAGAACTTGATTCACAAGGACTTGCAGAAATTGCAGTAGAAAGTGCAAAATCAGCATTA  **>tpi_4**  CACGAAACAGATGAAGAAATTAACAAAAAAGCGCACGCTATTTTCAAACATGGAATGACTCCAATTATATGTGTTGGTGAAACAGACGAAGAGCGTGAAAGTGGTAAAGCTAACGATGTTGTAGGTGAGCAAGTTAAGAAAGCTGTTGCAGGTTTATCTGAAGATCAACTTAAATCAGTTGTAATTGCTTATGAACCAATCTGGGCAATCGGAACTGGTAAATCATCAACATCTGAAGATGCAAATGAAATGTGTGCATTTGTACGTCAAACTATTGCTGACTTATCAAGCAAAGAAGTATCAGAAGCAACTCGTATTCAATATGGTGGTAGTGTTAAACCTAACAACATTAAAGAATACATGGCACAAACTGATATTGATGGGGCATTAGTAGGTGGCGCA  **>yqiL_3**  GCGTTTAAAGACGTGCCAGCCTATGATTTAGGTGCGACTTTAATAGAACATATTATTAAAGAGACGGGTTTGAATCCAAGTGAGATTGATGAAGTTATCATCGGTAACGTACTACAAGCAGGACAAGGACAAAATCCAGCACGAATTGCTGCTATGAAAGGTGGCTTGCCAGAAACAGTACCTGCATTTACGGTGAATAAAGTATGTGGTTCTGGGTTAAAGTCGATTCAATTAGCATATCAATCTATTGTGACTGGTGAAAATGACATCGTGCTAGCTGGCGGTATGGAGAATATGTCTCAATCACCAATGCTTGTCAACAACAGTCGCTTTGGTTTTAAAATGGGACATCAATCAATGGTTGATAGCATGGTATATGATGGTTTAACAGATGTATTTAATCAATATCATATGGGTATTACTGCTGAAAATTTAGTAGAGCAATATGGTATTTCAAGAGAAGAACAAGATACATTTGCTGTAAACTCACAACAAAAAGCAGTACGTGCACAGCAA |
| **239** | **>arcC_2**  TTATTAATCCAACAAGCTAAATCGAACAGTGACACAACGCCGGCAATGCCATTGGATACTTGTGGTGCAATGTCACAAGGTATGATAGGCTATTGGTTGGAAACTGAAATCAATCGCATTTTAACTGAAATGAATAGTGATAGAACTGTAGGCACAATCGTAACACGTGTGGAAGTAGATAAAGATGATCCACGATTTGATAACCCAACTAAACCAATTGGTCCTTTTTATACGAAAGAAGAAGTTGAAGAATTACAAAAAGAACAGCCAGGCTCAGTCTTTAAAGAAGATGCAGGACGTGGTTATAGAAAAGTAGTTGCGTCACCACTACCTCAATCTATACTAGAACACCAGTTAATTCGAACTTTAGCAGACGGTAAAAATATTGTCATTGCATGCGGTGGTGGCGGTATTCCAGTTATAAAAAAAGAAAATACCTATGAAGGTGTTGAAGCG  **>aroE_3**  AATTTTAATTCTTTAGGATTAGATGATACTTATGAAGCTTTAAATATTCCAATTGAAGATTTTCATTTAATTAAAGAAATTATTTCGAAAAAAGAATTAGAAGGCTTTAATATCACAATTCCTCATAAAGAACGTATCATACCGTATTTAGATTATGTTGATGAACAAGCGATTAATGCAGGTGCAGTTAACACTGTTTTGATAAAAGATGGCAAGTGGATAGGGTATAATACAGATGGTATTGGTTATGTTAAAGGATTGCACAGCGTTTATCCAGATTTAGAAAATGCATACATTTTAATTTTGGGCGCAGGTGGTGCAAGTAAAGGTATTGCTTATGAATTAGCAAAATTTGTAAAGCCCAAATTAACTGTTGCGAATAGAACGATGGCTCGTTTTGAATCTTGGAATTTAAATATAAACCAAATTTCATTAGCAGATGCTGAAAAGTATTTA  **>glpF_1**  GGTGCTGATTGGATTGTCATCACAGCTGGATGGGGATTAGCGGTTACAATGGGTGTGTTTGCTGTCGGTCAATTCTCAGGTGCACATTTAAACCCAGCGGTGTCTTTAGCTCTTGCATTAGACGGAAGTTTTGATTGGTCATTAGTTCCTGGTTATATTGTTGCTCAAATGTTAGGTGCAATTGTCGGAGCAACAATTGTATGGTTAATGTACTTGCCACATTGGAAAGCGACAGAAGAAGCTGGCGCGAAATTAGGTGTTTTCTCTACAGCACCGGCTATTAAGAATTACTTTGCCAACTTTTTAAGTGAGATTATCGGAACAATGGCATTAACTTTAGGTATTTTATTTATCGGTGTAAACAAAATTGCCGATGGTTTAAATCCTTTAATTGTCGGAGCATTAATTGTTGCAATCGGATTAAGTTTAGGCGGTGCTACTGGTTATGCAATCAACCCAGCACGT  **>gmk_1**  CGAATATTTGAAGATCCAAGTACATCATATAAGTATTCTATTTCAATGACAACACGTCAAATGCGTGAAGGTGAAGTTGATGGCGTAGATTACTTTTTTAAAACTAGGGATGCGTTTGAAGCTTTAATCAAAGATGACCAATTTATAGAATATGCTGAATATGTAGGCAACTATTATGGTACACCAGTTCAATATGTTAAAGATACAATGGACGAAGGTCATGATGTATTTTTAGAAATTGAAGTAGAAGGTGCAAAGCAAGTTAGAAAGAAATTTCCAGATGCGCTATTTATTTTCTTAGCACCTCCAAGTTTAGAACACTTGAGAGAGCGATTAGTAGGTAGAGGAACAGAATCTGATGAGAAAATACAAAGTCGTATTAACGAAGCGCGTAAAGAAGTTGAAATGATGAATTTA  **>pta_4**  GCAACACAATTACAAGCAACAGATTATGTTACACCAATCGTGTTAGGTGATGAGACTAAGGTTCAATCTTTAGCGCAAAAACTTGATCTTGATATTTCTAATATTGAATTAATTAATCCTGCGACAAGTGAATTGAAAGCTGAATTAGTTCAATCATTTGTTGAACGACGTAAAGGTAAAGCGACTGAAGAACAAGCACAAGAATTATTAAACAATGTGAACTACTTCGGTACAATGCTTGTTTATGCTGGTAAAGCAGATGGTTTAGTTAGTGGTGCAGCACATTCAACAGGCGACACTGTGCGTCCAGCTTTACAAATCATCAAAACGAAACCAGGTGTATCAAGAACATCAGGTATCTTCTTTATGATTAAAGGTGATGAACAATACATCTTTGGTGATTGTGCAATCAATCCAGAACTTGATTCACAAGGACTTGCAGAAATTGCAGTAGAAAGTGCAAAATCAGCATTA  **>tpi_4**  CACGAAACAGATGAAGAAATTAACAAAAAAGCGCACGCTATTTTCAAACATGGAATGACTCCAATTATATGTGTTGGTGAAACAGACGAAGAGCGTGAAAGTGGTAAAGCTAACGATGTTGTAGGTGAGCAAGTTAAGAAAGCTGTTGCAGGTTTATCTGAAGATCAACTTAAATCAGTTGTAATTGCTTATGAACCAATCTGGGCAATCGGAACTGGTAAATCATCAACATCTGAAGATGCAAATGAAATGTGTGCATTTGTACGTCAAACTATTGCTGACTTATCAAGCAAAGAAGTATCAGAAGCAACTCGTATTCAATATGGTGGTAGTGTTAAACCTAACAACATTAAAGAATACATGGCACAAACTGATATTGATGGGGCATTAGTAGGTGGCGCA  **>yqiL_3**  GCGTTTAAAGACGTGCCAGCCTATGATTTAGGTGCGACTTTAATAGAACATATTATTAAAGAGACGGGTTTGAATCCAAGTGAGATTGATGAAGTTATCATCGGTAACGTACTACAAGCAGGACAAGGACAAAATCCAGCACGAATTGCTGCTATGAAAGGTGGCTTGCCAGAAACAGTACCTGCATTTACGGTGAATAAAGTATGTGGTTCTGGGTTAAAGTCGATTCAATTAGCATATCAATCTATTGTGACTGGTGAAAATGACATCGTGCTAGCTGGCGGTATGGAGAATATGTCTCAATCACCAATGCTTGTCAACAACAGTCGCTTTGGTTTTAAAATGGGACATCAATCAATGGTTGATAGCATGGTATATGATGGTTTAACAGATGTATTTAATCAATATCATATGGGTATTACTGCTGAAAATTTAGTAGAGCAATATGGTATTTCAAGAGAAGAACAAGATACATTTGCTGTAAACTCACAACAAAAAGCAGTACGTGCACAGCAA |
| **8** | **>arcC_3**  TTATTAATCCAACAAGCTAAATCGAACAGTGACACAACGCCGGCAATGCCATTGGATACTTGTGGTGCAATGTCACAGGGTATGATAGGCTATTGGTTGGAAACTGAAATCAATCGCATTTTAACTGAAATGAATAGTGATAGAACTGTAGGCACAATCGTTACACGTGTGGAAGTAGATAAAGATGATCCACGATTTGATAACCCAACTAAACCAATTGGTCCTTTTTATACGAAAGAAGAAGTTGAAGAATTACAAAAAGAACAGCCAGACTCAGTCTTTAAAGAAGATGCAGGACGTGGTTATAGAAAAGTAGTTGCGTCACCACTACCTCAATCTATACTAGAACACCAGTTAATTCGAACTTTAGCAGACGGTAAAAATATTGTCATTGCATGCGGTGGTGGCGGTATTCCAGTTATAAAAAAAGAAAATACCTATGAAGGTGTTGAAGCG  **>aroE_3**  AATTTTAATTCTTTAGGATTAGATGATACTTATGAAGCTTTAAATATTCCAATTGAAGATTTTCATTTAATTAAAGAAATTATTTCGAAAAAAGAATTAGAAGGCTTTAATATCACAATTCCTCATAAAGAACGTATCATACCGTATTTAGATTATGTTGATGAACAAGCGATTAATGCAGGTGCAGTTAACACTGTTTTGATAAAAGATGGCAAGTGGATAGGGTATAATACAGATGGTATTGGTTATGTTAAAGGATTGCACAGCGTTTATCCAGATTTAGAAAATGCATACATTTTAATTTTGGGCGCAGGTGGTGCAAGTAAAGGTATTGCTTATGAATTAGCAAAATTTGTAAAGCCCAAATTAACTGTTGCGAATAGAACGATGGCTCGTTTTGAATCTTGGAATTTAAATATAAACCAAATTTCATTAGCAGATGCTGAAAAGTATTTA  **>glpF_1**  GGTGCTGATTGGATTGTCATCACAGCTGGATGGGGATTAGCGGTTACAATGGGTGTGTTTGCTGTCGGTCAATTCTCAGGTGCACATTTAAACCCAGCGGTGTCTTTAGCTCTTGCATTAGACGGAAGTTTTGATTGGTCATTAGTTCCTGGTTATATTGTTGCTCAAATGTTAGGTGCAATTGTCGGAGCAACAATTGTATGGTTAATGTACTTGCCACATTGGAAAGCGACAGAAGAAGCTGGCGCGAAATTAGGTGTTTTCTCTACAGCACCGGCTATTAAGAATTACTTTGCCAACTTTTTAAGTGAGATTATCGGAACAATGGCATTAACTTTAGGTATTTTATTTATCGGTGTAAACAAAATTGCCGATGGTTTAAATCCTTTAATTGTCGGAGCATTAATTGTTGCAATCGGATTAAGTTTAGGCGGTGCTACTGGTTATGCAATCAACCCAGCACGT  **>gmk_1**  CGAATATTTGAAGATCCAAGTACATCATATAAGTATTCTATTTCAATGACAACACGTCAAATGCGTGAAGGTGAAGTTGATGGCGTAGATTACTTTTTTAAAACTAGGGATGCGTTTGAAGCTTTAATCAAAGATGACCAATTTATAGAATATGCTGAATATGTAGGCAACTATTATGGTACACCAGTTCAATATGTTAAAGATACAATGGACGAAGGTCATGATGTATTTTTAGAAATTGAAGTAGAAGGTGCAAAGCAAGTTAGAAAGAAATTTCCAGATGCGCTATTTATTTTCTTAGCACCTCCAAGTTTAGAACACTTGAGAGAGCGATTAGTAGGTAGAGGAACAGAATCTGATGAGAAAATACAAAGTCGTATTAACGAAGCGCGTAAAGAAGTTGAAATGATGAATTTA  **>pta_4**  GCAACACAATTACAAGCAACAGATTATGTTACACCAATCGTGTTAGGTGATGAGACTAAGGTTCAATCTTTAGCGCAAAAACTTGATCTTGATATTTCTAATATTGAATTAATTAATCCTGCGACAAGTGAATTGAAAGCTGAATTAGTTCAATCATTTGTTGAACGACGTAAAGGTAAAGCGACTGAAGAACAAGCACAAGAATTATTAAACAATGTGAACTACTTCGGTACAATGCTTGTTTATGCTGGTAAAGCAGATGGTTTAGTTAGTGGTGCAGCACATTCAACAGGCGACACTGTGCGTCCAGCTTTACAAATCATCAAAACGAAACCAGGTGTATCAAGAACATCAGGTATCTTCTTTATGATTAAAGGTGATGAACAATACATCTTTGGTGATTGTGCAATCAATCCAGAACTTGATTCACAAGGACTTGCAGAAATTGCAGTAGAAAGTGCAAAATCAGCATTA  **>tpi_4**  CACGAAACAGATGAAGAAATTAACAAAAAAGCGCACGCTATTTTCAAACATGGAATGACTCCAATTATATGTGTTGGTGAAACAGACGAAGAGCGTGAAAGTGGTAAAGCTAACGATGTTGTAGGTGAGCAAGTTAAGAAAGCTGTTGCAGGTTTATCTGAAGATCAACTTAAATCAGTTGTAATTGCTTATGAACCAATCTGGGCAATCGGAACTGGTAAATCATCAACATCTGAAGATGCAAATGAAATGTGTGCATTTGTACGTCAAACTATTGCTGACTTATCAAGCAAAGAAGTATCAGAAGCAACTCGTATTCAATATGGTGGTAGTGTTAAACCTAACAACATTAAAGAATACATGGCACAAACTGATATTGATGGGGCATTAGTAGGTGGCGCA  **>yqiL_3**  GCGTTTAAAGACGTGCCAGCCTATGATTTAGGTGCGACTTTAATAGAACATATTATTAAAGAGACGGGTTTGAATCCAAGTGAGATTGATGAAGTTATCATCGGTAACGTACTACAAGCAGGACAAGGACAAAATCCAGCACGAATTGCTGCTATGAAAGGTGGCTTGCCAGAAACAGTACCTGCATTTACGGTGAATAAAGTATGTGGTTCTGGGTTAAAGTCGATTCAATTAGCATATCAATCTATTGTGACTGGTGAAAATGACATCGTGCTAGCTGGCGGTATGGAGAATATGTCTCAATCACCAATGCTTGTCAACAACAGTCGCTTTGGTTTTAAAATGGGACATCAATCAATGGTTGATAGCATGGTATATGATGGTTTAACAGATGTATTTAATCAATATCATATGGGTATTACTGCTGAAAATTTAGTAGAGCAATATGGTATTTCAAGAGAAGAACAAGATACATTTGCTGTAAACTCACAACAAAAAGCAGTACGTGCACAGCAA |
| **772** | **>arcC_1**  TTATTAATCCAACAAGCTAAATCGAACAGTGACACAACGCCGGCAATGCCATTGGATACTTGTGGTGCAATGTCACAGGGTATGATAGGCTATTGGTTGGAAACTGAAATCAATCGCATTTTAACTGAAATGAATAGTGATAGAACTGTAGGCACAATCGTTACACGTGTGGAAGTAGATAAAGATGATCCACGATTCAATAACCCAACCAAACCAATTGGTCCTTTTTATACGAAAGAAGAAGTTGAAGAATTACAAAAAGAACAGCCAGACTCAGTCTTTAAAGAAGATGCAGGACGTGGTTATAGAAAAGTAGTTGCGTCACCACTACCTCAATCTATACTAGAACACCAGTTAATTCGAACTTTAGCAGACGGTAAAAATATTGTCATTGCATGCGGTGGTGGCGGTATTCCAGTTATAAAAAAAGAAAATACCTATGAAGGTGTTGAAGCG  **>aroE_1**  AATTTTAATTCTTTAGGATTAGATGATACTTATGAAGCTTTAAATATTCCAATTGAAGATTTTCATTTAATTAAAGAAATTATTTCGAAAAAAGAATTAGATGGCTTTAATATCACAATTCCTCATAAAGAACGTATCATACCGTATTTAGATCATGTTGATGAACAAGCGATTAATGCAGGTGCAGTTAACACTGTTTTGATAAAAGATGACAAGTGGATAGGGTATAATACAGATGGTATTGGTTATGTTAAAGGATTGCACAGCGTTTATCCAGATTTAGAAAATGCATACATTTTAATTTTGGGCGCAGGTGGTGCAAGTAAAGGTATTGCTTATGAATTAGCAAAATTTGTAAAGCCCAAATTAACTGTTGCGAATAGAACGATGGCTCGTTTTGAATCTTGGAATTTAAATATAAACCAAATTTCATTAGCAGATGCTGAAAAGTATTTA  **>glpF_1**  GGTGCTGATTGGATTGTCATCACAGCTGGATGGGGATTAGCGGTTACAATGGGTGTGTTTGCTGTCGGTCAATTCTCAGGTGCACATTTAAACCCAGCGGTGTCTTTAGCTCTTGCATTAGACGGAAGTTTTGATTGGTCATTAGTTCCTGGTTATATTGTTGCTCAAATGTTAGGTGCAATTGTCGGAGCAACAATTGTATGGTTAATGTACTTGCCACATTGGAAAGCGACAGAAGAAGCTGGCGCGAAATTAGGTGTTTTCTCTACAGCACCGGCTATTAAGAATTACTTTGCCAACTTTTTAAGTGAGATTATCGGAACAATGGCATTAACTTTAGGTATTTTATTTATCGGTGTAAACAAAATTGCCGATGGTTTAAATCCTTTAATTGTCGGAGCATTAATTGTTGCAATCGGATTAAGTTTAGGCGGTGCTACTGGTTATGCAATCAACCCAGCACGT  **>gmk_1**  CGAATATTTGAAGATCCAAGTACATCATATAAGTATTCTATTTCAATGACAACACGTCAAATGCGTGAAGGTGAAGTTGATGGCGTAGATTACTTTTTTAAAACTAGGGATGCGTTTGAAGCTTTAATCAAAGATGACCAATTTATAGAATATGCTGAATATGTAGGCAACTATTATGGTACACCAGTTCAATATGTTAAAGATACAATGGACGAAGGTCATGATGTATTTTTAGAAATTGAAGTAGAAGGTGCAAAGCAAGTTAGAAAGAAATTTCCAGATGCGCTATTTATTTTCTTAGCACCTCCAAGTTTAGAACACTTGAGAGAGCGATTAGTAGGTAGAGGAACAGAATCTGATGAGAAAATACAAAGTCGTATTAACGAAGCGCGTAAAGAAGTTGAAATGATGAATTTA  **>pta_22**  GCAACACAATTACAAGCAACAGATTATGTTACACCAATCGTGTTAGGTGATGAGACTAAGGTTCAATCTTTAGCGCAAAAACTTGATCTTGATATTTCTAATATTGAATTAATTAATCCTGCGACAAGTGAATTGAAAGCTGAATTAGTTCAATCATTTGTTGAACGACGTAAAGGTAAAGCGACTGAAGAACAAGCACAAGAATTATTAAACAATGTGAACTACTTCGGTACAATGCTTGTTTATGCTGGTAAAGCAGATGGTTTAGTTAGTGGTGCAGCACATTCAACAGGAGACACTGTGCGTCCAGCTTTACAAATCATCAAAACGAAACCAGGTGTATCAAGAACATCAGGTATCTTCTTTATGATTAAAGGTGATGAACAATACATCTTTGGCGATTGTGCAATCAATCCAGAACTTGATTCACAAGGACTTGCAGAAATTGCAGTAGAAAGTGCAAAATCAGCATTA  **>tpi_1**  CACGAAACAGATGAAGAAATTAACAAAAAAGCGCACGCTATTTTCAAACATGGAATGACTCCAATTATTTGTGTTGGTGAAACAGACGAAGAGCGTGAAAGTGGTAAAGCTAACGATGTTGTAGGTGAGCAAGTTAAGAAAGCTGTTGCAGGTTTATCTGAAGATCAACTTAAATCAGTTGTAATTGCTTATGAGCCAATCTGGGCAATCGGAACTGGTAAATCATCAACATCTGAAGATGCAAATGAAATGTGTGCATTTGTACGTCAAACTATTGCTGACTTATCAAGCAAAGAAGTATCAGAAGCAACTCGTATTCAATATGGTGGTAGTGTTAAACCTAACAACATTAAAGAATACATGGCACAAACTGATATTGATGGGGCATTAGTAGGTGGCGCA  **>yqiL_1**  GCGTTTAAAGACGTGCCAGCCTATGATTTAGGTGCGACTTTAATAGAACATATTATTAAAGAGACGGGTTTGAATCCAAGTGAGATTGATGAAGTTATCATCGGTAACGTACTACAAGCAGGACAAGGACAAAATCCAGCACGAATTGCTGCTATGAAAGGTGGCTTGCCAGAAACAGTACCTGCATTTACAGTGAATAAAGTATGTGGTTCTGGGTTAAAGTCGATTCAATTAGCATATCAATCTATTGTGACTGGTGAAAATGACATCGTGCTAGCTGGCGGTATGGAGAATATGTCTCAGTCACCAATGCTTGTCAACAACAGTCGCTTCGGTTTTAAAATGGGACATCAATCAATGGTTGATAGCATGGTATATGATGGTTTAACAGATGTATTTAATCAATATCATATGGGTATTACTGCTGAAAATTTAGTGGAGCAATATGGTATTTCAAGAGAAGAACAAGATACATTTGCTGTAAACTCACAACAAAAAGCAGTACGTGCACAGCAA |
